# A comparative analysis of current phasing and imputation software

**DOI:** 10.1371/journal.pone.0260177

**Published:** 2022-10-19

**Authors:** Adriano De Marino, Abdallah Amr Mahmoud, Madhuchanda Bose, Karatuğ Ozan Bircan, Andrew Terpolovsky, Varuna Bamunusinghe, Sandra Bohn, Umar Khan, Biljana Novković, Puya G. Yazdi

**Affiliations:** Research & Development, SelfDecode, Miami, FL, United States of America; University of North Carolina at Chapel Hill, UNITED STATES

## Abstract

Whole-genome data has become significantly more accessible over the last two decades. This can largely be attributed to both reduced sequencing costs and imputation models which make it possible to obtain nearly whole-genome data from less expensive genotyping methods, such as microarray chips. Although there are many different approaches to imputation, the Hidden Markov Model (HMM) remains the most widely used. In this study, we compared the latest versions of the most popular HMM-based tools for phasing and imputation: Beagle5.4, Eagle2.4.1, Shapeit4, Impute5 and Minimac4. We benchmarked them on four input datasets with three levels of chip density. We assessed each imputation software on the basis of accuracy, speed and memory usage, and showed how the choice of imputation accuracy metric can result in different interpretations. The highest average concordance rate was achieved by Beagle5.4, followed by Impute5 and Minimac4, using a reference-based approach during phasing and the highest density chip. IQS and R^2^ metrics revealed that Impute5 and Minimac4 obtained better results for low frequency markers, while Beagle5.4 remained more accurate for common markers (MAF>5%). Computational load as measured by run time was lower for Beagle5.4 than Minimac4 and Impute5, while Minimac4 utilized the least memory of the imputation tools we compared. ShapeIT4, used the least memory of the phasing tools examined with genotype chip data, while Eagle2.4.1 used the least memory phasing WGS data. Finally, we determined the combination of phasing software, imputation software, and reference panel, best suited for different situations and analysis needs and created an automated pipeline that provides a way for users to create customized chips designed to optimize their imputation results.

## Introduction

Genome wide association studies (GWAS) remain one of the most critical and powerful methods of identifying key genes and variants that play a role in many common human diseases [1, 2]. Identification of disease-associated variants in GWAS is dependent on successful tagging of millions of common variants in the human genome, and the ability to make inferences about genotypes of rare variants which are often not in linkage disequilibrium (LD) with common variants [1, 2]. Commercial single nucleotide polymorphism (SNP) genotyping arrays can contain up to 2.5 million markers, but none provide complete coverage of the human genome [3]. Despite the advances of the last two decades which have led to increasingly rapid and extensive genotyping, it is still prohibitively expensive to obtain whole genome sequencing (WGS) for the tens of thousands of individuals in GWAS [4, 5]. Individual GWAS may also use distinct chips with different markers. To combine these GWAS for meta analysis, we require a method by which to identify genotypes at all markers utilized in each of these studies [6]. Thus, we continue to rely on imputation, the process of probabilistically estimating non-genotyped alleles for individuals in GWAS samples.

**Genotype imputation** is a method that infers the alleles of un-genotyped single-nucleotide polymorphisms (SNPs) based on linkage disequilibrium (LD) with directly genotyped markers using a suitable reference population [7]. It is predicated on the idea that seemingly unrelated individuals from the human population sampled at random can share short stretches of DNA within chromosomes derived from a shared ancestor [8]. Imputation can be used to improve SNP coverage and increase the statistical power of GWAS [9, 10]. Genotype imputation also facilitates fine mapping of causal variants, plays a key role in the meta-analyses of GWAS, and can be utilized in downstream applications of GWAS such as estimation of disease risk [9]. However, an important limitation of imputation is that only variants that were previously observed in a reference panel can be imputed [9]. Furthermore, rare variants are often poorly represented in reference panels making accurate imputation of rare and infrequent variants difficult. In addition, the choice of whether to pre-phase the data can impact imputation. Finally, imputation accuracy, sensitivity and computational efficiency are greatly affected by the choice of imputation software or tool [9].

Over the last twenty years, multiple research groups have developed and published a number of phasing and imputation models, the majority of which are based on the Li and Stephens Hidden Markov Model (HMM) [10]. First described in 2003, it was applied to haplotype estimation methods, termed "phasing", and used to handle large stretches of chromosome where individual haplotypes share contiguous, mosaic stretches with other haplotypes in the sample [8, 9]. Unlike previous coalescent approaches, it was computationally tractable, and methods based on the Li & Stephens HMM were soon shown to be more accurate and efficient than other methods [8, 11]. Landmark and popular phasing algorithms are listed in Table 1, as a brief tabular history of the field. Currently, the most commonly used Li and Stephens HMM-based software’s are BEAGLE, EAGLE, and SHAPEIT for phasing, and BEAGLE, IMPUTE and MINIMAC for imputation.

**Table 1:** A brief history of phasing and imputation tools.

	Software	Published	Based on	Features	Complexity
**Phasing**	PHASE v 1.0 [12]	2001	Coalescent approximation	Improved error rates are reduced by >50% relative to its nearest competitor	quadratic O(n^2^)
	HAPI-UR [13]	2012	Li & Stephens HMM	Used windows of sites instead of specific markers; led to higher accuracy	linear O(nm)
	Eagle 2 [14]	2016	Li & Stephens HMM	pBWT on a large reference panel condensed into a set of compact tree structures that losslessly model haplotype structure	linear O(nm)
**Phasing & Imputation**	fastPHASE [8]	2006	Li & Stephens HMM	Faster but less accurate than Phase	linear O(nm)
	Beagle v. 1.0 [15]	2007	Li & Stephens HMM	Uses bifurcating tree structure (aka haplotype-cluster model)	quadratic O(n^2^)
	Beagle v. 2.0, 3.0 [16, 17]	2009	Li & Stephens HMM	Uses bifurcating tree structure (aka haplotype-cluster model)	quadratic O(n^2^)
	Beagle v. 4.0 [18]	2018	Li & Stephens HMM	Abandoned bifurcating model to adopt a flexible choice of haplotypes for reference similar to IMPUTE 2	quadratic O(n^2^)
	Beagle v. 5.2 [19]	2021	Li & Stephens HMM	Introduction of progressive phasing algorithm to handle hundreds of millions of markers	linear O(nm)
	IMPUTE 2 [20]	2009	Li & Stephens HMM	Flexible choice of haplotypes for reference panel; quadratic computational complexity meant inefficient	linear O(nm)
	IMPUTE 4 [21]	2018	Li & Stephens HMM	Speed up haplotype imputation step	quadratic O(n^2^)
	IMPUTE 5 [22]	2019	Li & Stephens HMM	Uses positional BWT to choose haplotypes for each window	linear O(nm)
	MACH [23]	2010	Li & Stephens HMM	An iteratively updated phase of each study sample	linear O(m+n)
	SHAPEIT 1 [24]	2011	Li & Stephens HMM	Flexible choice of the panel but computationally efficient	linear O(n+m)
	SHAPEIT 2 [25]	2013	Li & Stephens HMM	Combined best aspects of SHAPEIT 1 and IMPUTE 2 to increase accuracy and efficiency	quadratic O(mn^2^)
	SHAPEIT 3 [26]	2016	Li & Stephens HMM	Increased scalability from SHAPEIT 2	quadratic O(n^2^)
	SHAPEIT 4 [27]	2018	Li & Stephens HMM	pBWT to choose haplotypes for local window	linear O(nm)
**Imputation**	Minimac [28]	2012	Li & Stephens HMM	Pre-phased imputation	linear O(nm)
	Minimac 2 [29]	2014	Li & Stephens HMM	Improved version and bug fixing	linear O(nm)
	Minimac 3 [30]	2015	Li & Stephens HMM	State-space reduction to reduce computational complexity and cost	linear O(nm)
	Minimac4 [31]	2018	Li & Stephens HMM	Improved version and bug fixing	linear O(nm)

A timeline and brief description of landmark and popular phasing and imputation algorithms and their computational complexities

Imputation accuracy is measured by several key sets of metrics which can be classified into two overarching types: statistics that compare imputed genotypes to ‘gold standard’ genotyped data and statistics produced without reference to true genotypes [32]. Concordance rate, squared correlation (R^2^), and Imputation Quality Score (IQS) are examples of the first type [32, 33]. In practice, the purpose of imputation is to predict SNPs for which we do not have genotyped data; statistics of the second type are typically relied upon during imputation, and generally output by the various imputation programs. Although the rapid increase in the number of deeply sequenced individuals will soon make it possible to assemble increasingly large reference panels that greatly increase the number of imputable variants, the choice of phasing and imputation software currently has a significant impact on accuracy [34].

While several studies have evaluated and compared imputation models, or phasing models, or imputation models in combination with different reference panels, no recent studies have compared imputation and phasing algorithms in combination with different reference panels and datasets, in tandem, and evaluated the relative computational efficiency and accuracy of each combination [34, 35]. Previous studies which have examined differences between phasing and imputation tools have worked with earlier iterations of these or similar tools. A 2018 comparison of phasing tools by Choi et al. [36] examined switch error rates and percent of variants phased using either the Haplotype Reference Consortium or the 1000 Genomes Project as a reference panel, by then state-of-the-field tools: Eagle2, SHAPEIT, Beagle, Illumina’s HapCUT, and less popular tools such as CPT, Moleculo and Fosmid [36].

In this study, we evaluate the latest versions of the most commonly used tools for phasing and imputation in terms of accuracy, computational speed and memory usage, using 2 different versions of the 1000 Genome Project as reference panels and four different microarray chip datasets as inputs (S1 Table). We combine each tool for phasing with a method for imputation to understand which combination achieves the best overall results and which method is the best at imputing rare variants. Our goal was to determine the combination of phasing and imputation software and reference panel that is best suited for different situations and needs.

## Methods

### Data

We used four different chip datasets, with differing marker density and input dataset sizes. The first chip dataset Estonian Biobank (EBB) was composed of 2280 unrelated individuals, a whole genome sequencing (WGS) dataset converted into chip data, EBB is a volunteer-based sample of the Estonian resident adult population (aged ≥18 years) (dbGAP Accession Number: phs001230.v1.p1). The second (Affymetrix) was composed of 3450 unrelated individuals from The 1000 Genomes Project genotyped with the Affymetrix 6.0 900K array (Affymetrix, ThermoFisher), the third one (Omni) of 2318 unrelated individuals from the 1000 Genomes Project genotyped with the Omni 2.5 chip by Illumina 2.4 Million unphased SNP markers, and the forth one (Customized) was a subset of the Affymetrix and Omni chip and consisted of the intersection of the Affymetrix and Omni chips with another chip, GSA version 3 with direct-to-consumer booster by Illumina (S1 Fig). This Customized chip is the intersection of commonly used chips, resulting in a low-density chip with fewer overall sites, to allow us to assess imputation and phasing accuracy when the input data is limited to a relatively small number of SNPs.

**Fig 1:**
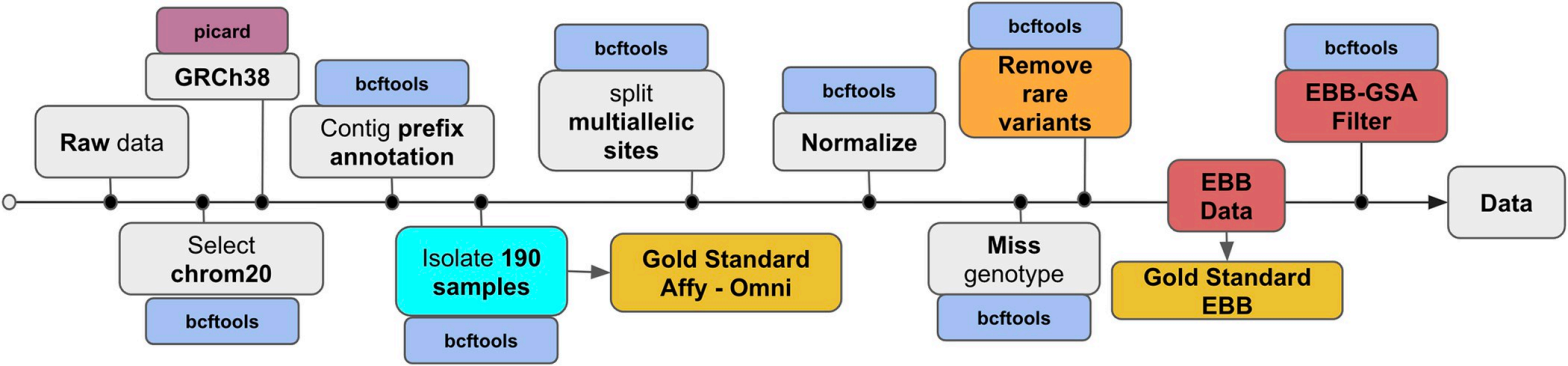
Pre-processing of the HD genotype chips, reference panels and WGS EBB data. Pre-processing of the HD genotype chips, reference panels and WGS EBB data downloaded from the International Genome Sample Resource (IGSR) and Estonian Biobank Estonian Genome Center respectively. Steps highlighted in orange are specific to the 1000GPphase3 reference panel only; steps highlighted in red are specific to EBB data only and steps highlighted in cyan (light blue) are specific for chip Affymetrix and Omni to isolate only a portion of the dataset to perform analysis on it. All other steps were performed for both reference panels and datasets.

Data called from EBB, Affymetrix and Omni data were normalized using BCFtools [37]. The resulting chip data was processed separately for each chromosome. Chromosome 20 was chosen for use in all downstream analyses as it is generally representative of autosomal chromosomes. Sample data were converted to GRCh38 with Picard Toolkit. 2019. Broad Institute. GitHub Repository. https://broadinstitute.github.io/picard, to match the reference panels, multiallelic sites were split, variants left-normalized to the reference genome, and duplicate variants removed (Fig 1). Finally, because Beagle does not allow skipping imputation of sporadic missing data, variants with missing genotype information were removed from both the chip datasets, the WGS EBB data and the reference panels.

Finally, we converted the WGS EBB data (1,071,486 variants for chr20) in chip genotype size using a variant filtering with GSA chip data (15,635 variants for chr20), we kept only the variants in common, resulting in 13,990 variants left for chr20. We will refer to this new dataset as EBB chip data.

### Reference panel collection and sample selection

We drew our reference panels for imputation and phasing from the “The 1000 Genomes Project” (1000GP). We used the phase 3 low coverage WGS which has a mean depth of 7X as one reference panel and the high coverage WGS, with a mean depth of 30x, as a second reference panel [38, 39]https://www.zotero.org/google-docs/?broken=OoRgs5. We refer to these as the 1000GP-Phase3 and 1000GP-30x reference panels.

We selected 2280 unrelated individuals from the EBB collection. Imputation accuracy was assessed by looking at the concordance between the imputed EBB chip data and the whole genome sequences for these 2280 samples from the original WGS EBB dataset.

Further, in order to test imputation accuracy between different populations, we randomly selected 190 unrelated individuals (S2 Fig) taken from the set of 1686 individuals found in all three collections—the Omni, Affymetrix and WGS 1000 Genomes Project sample collections [39] as shown in S2 Fig. Our sample consisted of 5 males and 5 females per population, for 19 different populations and 5 super-populations (S3 Fig). These 190 individuals, and their relatives, were removed from the reference panels and used to create chip datasets for testing. Imputation accuracy was assessed by looking at the concordance between the imputed chips’ data and the whole genome sequences for these 190 samples.

### Quality control of reference panels

For both 1000GP reference panels and EBB data, we used BCFtools [37] to split multiallelic sites, remove duplicates and missing data, and align variants to the reference genome. Both the 1000GP-30x and 1000GP-Phase3 panels were preprocessed by prepending the contig name with the prefix ‘chr’. We created another 1000GP-30x where filtered out all the non-common variants that weren’t inside the WGS EBB data because imputation accuracy could not be assessed for those. Two additional steps were performed for the 1000GP-Phase3 panel to convert it to GRCh38 with Picard liftover, and discard rare variants singletons and doubletons to evaluate if their removal increased imputation accuracy for common variants (MAF>5%). This last operation was done only for the reference panel in chip data Affymetrix, Omni, Customized, while for EBB data we kept all the variants in common between these 2 reference panels and looked at the imputation accuracy differences between 1000GP-Phase3 and 1000GP-30x. The workflow for the quality control and pre-processing of the reference panels is shown in Fig 1.

### Phasing and imputation pipeline

**Fig 2:**
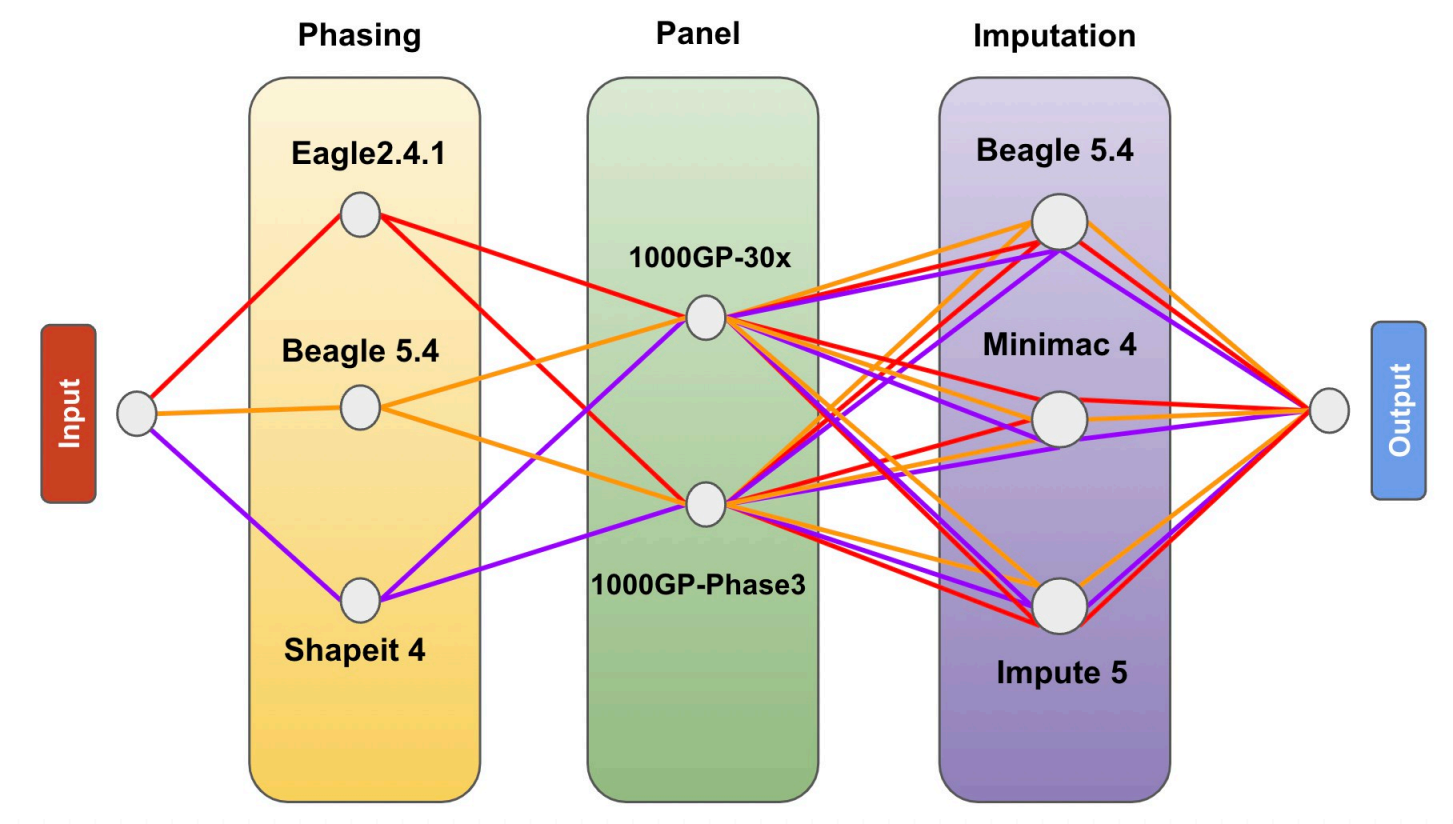
Workflow of the analysis, combinations tested. Affymetrix, Omni, Customized, and EBB input chip datasets were analyzed using the 36 combinations of 3 different phasing software, 2 phasing approaches, 3 imputation software, and 2 imputation reference panels. EBB input chip dataset was analyzed using the 36 combinations of 3 different phasing software, 2 Reference panels, 2 phasing approaches and 3 imputation software.

The EBB, Affymetrix, Omni and Customized chips were used as inputs for 9 combinations of phasing and imputation tools to assess which combination performed best for our sample set (Fig 2), using one of the two reference panels. Phasing was performed using both reference-free and reference-based approaches for each method, to compare their respective imputation accuracy. This yielded a total of 144 combinations of 4 input chip datasets, 3 phasing tools, reference-based or reference-free phasing approach, 2 imputation reference panels, and 3 imputation tools (S1 Table).

The haplotype phasing software we compared are: Eagle2 v2.4.1 [14], Beagle5 v5.4 [18], and Shapeit4 v4.2.1 [27]. All phasing software was launched with default parameters using 4 cores for each analysis on an Intel Corporation 82371AB/EB/MB PIIX4 ACPI 64-bit 32Gb RAM and the saved log file was used to evaluate the total run time. The imputation methods we tested are: Beagle5 v5.4 [18], Impute5 v1.1.5 [22] and Minimac4 v1.0.0 [30].

Each input chip dataset was processed using **Imputation_score.sh** an automated pipeline we built in bash that combines the phasing and imputation software and evaluates accuracy at each step to speed up the process of analysis and comparison. The inputs to the pipeline are the chip data file, a reference panel, the number of threads to use and the chromosome to process. The pipeline first checks that the correct version of the reference panel already exists for each imputation software and if the input file is available both in BCF format and in VCF format. This means that the original reference panel is converted to **bref3** for Imputation with Beagle5.4 using *bref3*.*29May21*.*d6d*.*jar*, to **m3mv** for Minimac4 using Minimac3 and to **imp5** for Impute5 using imp5Converter_1.1.5_static. If any of these files don’t exist, they are automatically created by the pipeline. After this initial check, the pipeline begins phasing the haplotypes using Eagle2.4.1, Beagle5.4 and Shapeit4. Each of these softwares was run twice with default parameters, once with the reference panel and once without, using 4 threads on chromosome 20 with recombination rates drawn from the genetic map. This step generated 2 phased VCF files for each software, yielding a total of 6 phased VCF files. After phasing, VCF files were moved to imputation with Beagle5.4, Minimac4 and Impute5. All were run using default parameters with a genetic map for the recombination rate and 4 threads. There are options to speed up both Minimac4 and Impute5, but these tend to reduce the accuracy rate. To maximize the accuracy of each tool and preserve the validity of the comparison, we ran them with the default parameters, avoiding the steps required to optimize for computational load.

### Accuracy measurement

Imputation accuracy was assessed by comparing the imputation data resulting from each of the different combinations of phasing tool, imputation tool, and choice of reference, against the WGS dataset of the chosen 190 target samples for Affymetrix Omni and customized chip and against the WGS EBB dataset of the chosen 2280 target samples for the EBB chip data. Variables considered were population/ancestry, sex, choice of tools, choice of reference, use of a reference panel, chip density, and the effect of MAF. We also looked at computational efficiency and memory usage. To check the effects of MAF on imputation accuracy, we used R^2^ as the metric of choice as it can distinguish between different MAF stratifications and is the most widely used metric for assessing imputation accuracy [40]. We also used IQS [32].

Phasing accuracy was evaluated using 540 children from the 1000GP-30x reference panel. These 540 children were phased using trioPhaser, a mendelian inheritance logic, to improve genomic haplotypes phasing. To ensure the greatest possible phasing accuracy, trioPhaser phases by parent’s genomes (mother and father) to identify switch errors by comparing the phasing of the children against the phased parent chromosomes; for a total of 1620 individuals analyzed. These 540 phased children have been used as a ground truth set to determine phasing accuracy in our analysis. In addition, a new reference panel (non-representative reference panel) was generated to assess reference-based phasing performance against the reference-free approach. It was composed of 2280 individuals from the Estonian BioBank and all unrelated individuals from the 1000GP-30x (932 individuals), for a total of 3212 individuals and 502,377 variants. Only the variants in common between EBB and 1000GP-30x were selected, in order to assess the phasing accuracy.

Imputation and phasing accuracy were evaluated using a custom, faster version of the imputation accuracy calculation software available on Github the accuracy metrics described in the work of Ramnarine et al. 2015 [32]. A detailed report with the concordance ratio (Po), F-measure score, square correlation (R^2^) and imputation quality score (IQS) was generated and written to the output file. To accurately assess IQS and R^2^ results, we removed all variants with MAF equal to 0 in our target population (allele count equal to 0) of 2280 individuals from the analysis; IQS is zero when MAF is equal to zero and is not indicative of accuracy or imputation quality. The entire code for accuracy metrics can be found in the script Simpy.py (section Data Available).

## Results

### Genotyping data

**Fig 3:**
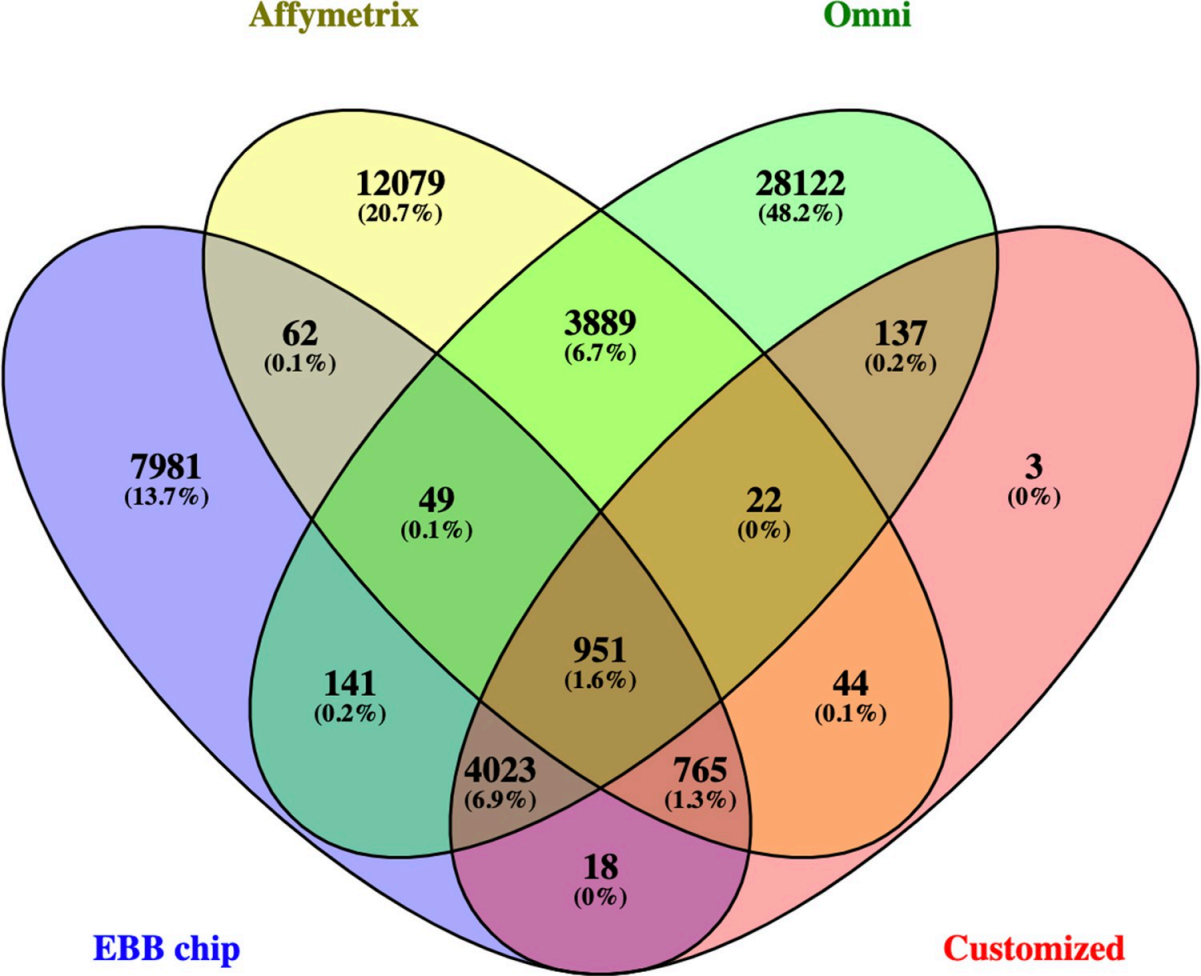
Number of shared variants between datasets. Variants in common between the different chips on chromosome 20.

After performing quality control on chromosome 20, 13,990 variants with a genotyping call rate of 100% remained in the EBB chip dataset, 17,861 variants with a genotyping call rate of 100% remained in the Affymetrix chip dataset, and 37,334 variants with a genotyping call rate of 100% remained in the Omni Illumina dataset. In total, 4,911 SNP markers overlapped between Omni and Affymetrix chips. The customized chip had 5963 markers shared between the GSA and the Affymetrix and Omni chips. The number of variants shared between the chip datasets is shown in Fig 3.

### Phasing

#### Accuracy

**Fig 4:**
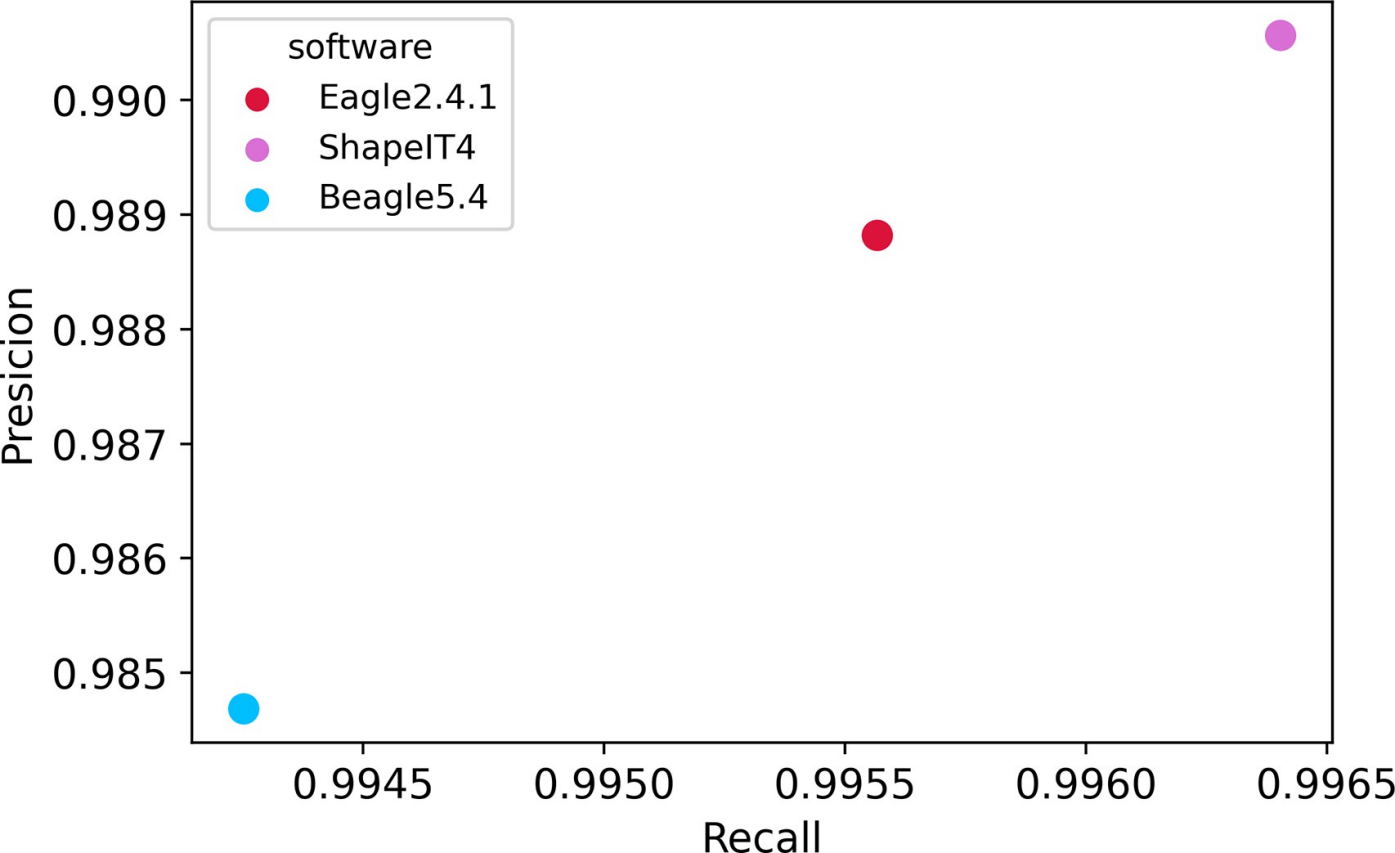
Precision and recall evaluation of phasing softwares Beagle5.4, ShapeIT4, and Eagle2.4.1. Precision and recall were evaluated using 540 trio children in the 1000GP-30x reference panel. Trios were selected and phased using trioPhaser software to ensure the highest accuracy and then the children were used as ground truth for the comparison. ShapeIT4 (pink dot) got the highest scores over Eagle2.4.1 and Beagle5.4 respectively.

The phasing accuracy has been evaluated using 540 children coming from trios in the 1000GP-30x reference panel. We calculated precision and recall to determine which haplotype estimation software was the most accurate amongst the three we compared: Beagle5.4, ShapeIT4, and Eagle2.4.1. The results were compared with those from the trioPhaser software. ShapeIT4 had the highest accuracy, on average, with 0.991 precision and 0.9964 recall. On the other hand, Beagle5.4 had the lowest precision at 0.9848, and 0.9942 recall, on average. Eagle2.4.1 was in the middle with 0.98899 precision and 0.9958 recall (Fig 4).

We tested the effect of using a non-representative reference panel on phasing accuracy to compare against reference-free phasing in the hypothetical scenario where a representative panel is not available. Non-representative reference panel was used to phase trio children present in the same release of the 1000GP. In this test case, using a non-representative reference panel, we found that reference-free phasing accuracy was higher than reference-based phasing (Table 2).

**Table 2:** Reference-free and reference-based phasing accuracy based on 502,377 variants.

Method	Phasing Software	Accuracy %
**Reference Based**	**Beagle5.4**	93.400
	**Eagle2.4.1**	93.554
	**ShapeIT4**	93.597
**Reference Free**	**Beagle5.4**	94.176
	**Eagle2.4.1**	94.164
	**ShapeIT4**	94.154

#### Speed and memory usage in phasing

**Fig 5:**
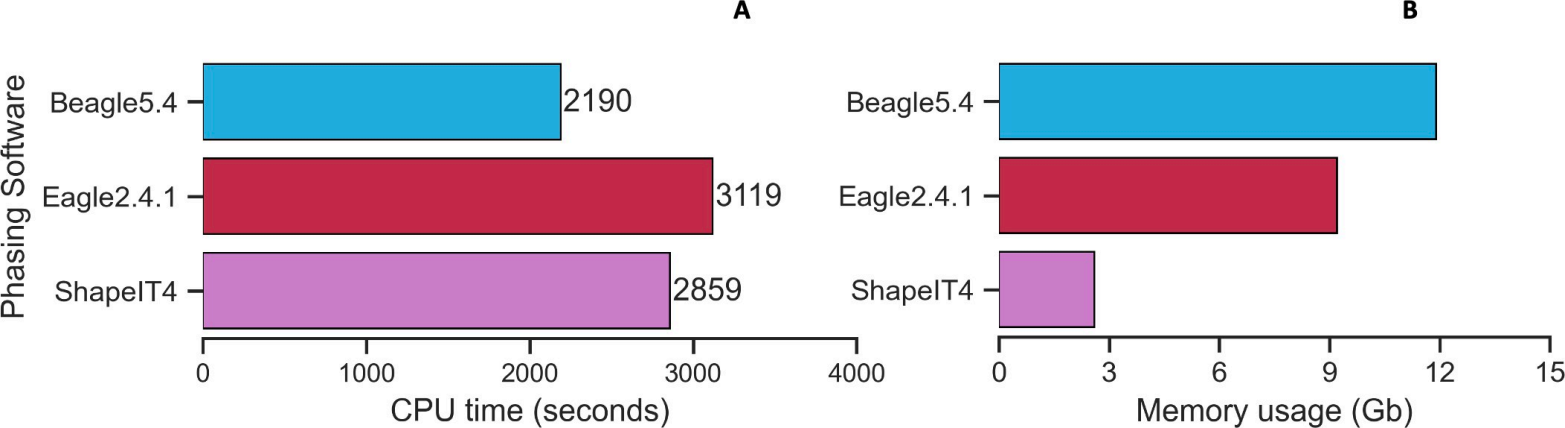
CPU run time and memory usage of phasing software in trios dataset. Average run time for phasing (5A). Average memory usage for phasing (5B) in trios data.

The phasing of the 540 children coming from trios in the 1000GP-30x reference panel required on average 2722 secs (~45mins) CPU time (Fig 5A) and ~8 Gb of memory usage (Fig 5B). During phasing, Eagle2.4.1 and ShapeIT4 used less memory than Beagle5.4, while Beagle5.4 was faster.

**Fig 6:**
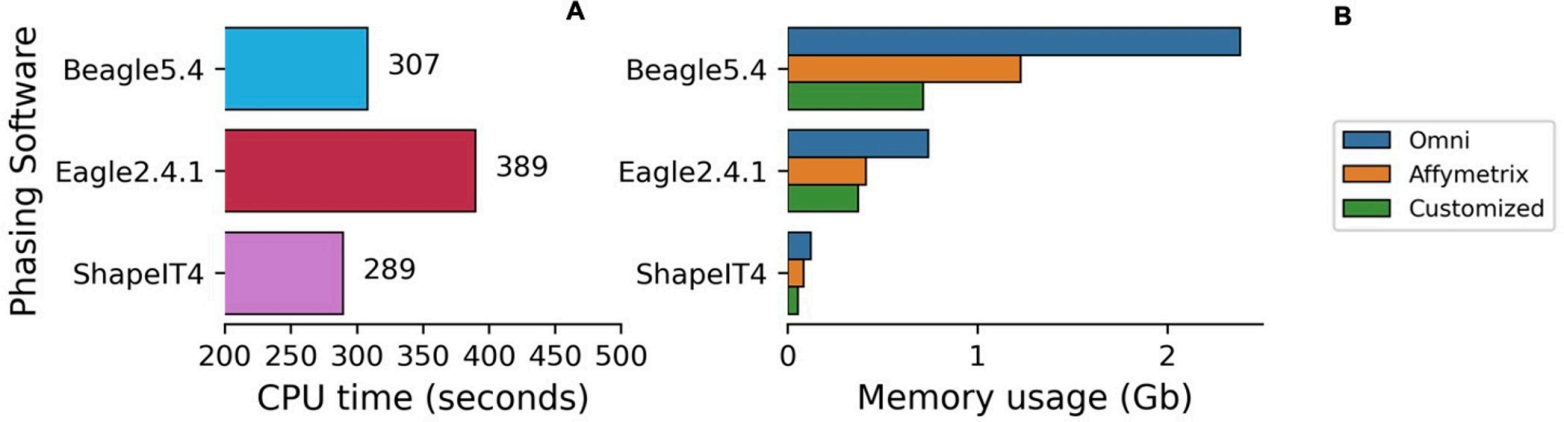
CPU run time and memory usage of phasing software using chip Omni, Affymetrix and Customized. Average run time for phasing (6A). Average memory usage for phasing (6B) in chips data.

In Affymetrix, Omni and Customized chip data during phasing, Eagle2.4.1 and ShapeIT4 used less memory than Beagle5.4 and were less affected by the input size of the chip (Fig 6). Averaged across the datasets, Eagle2.4.1 was the slowest phasing software while ShapeIT4 was the fastest.

**Fig 7:**
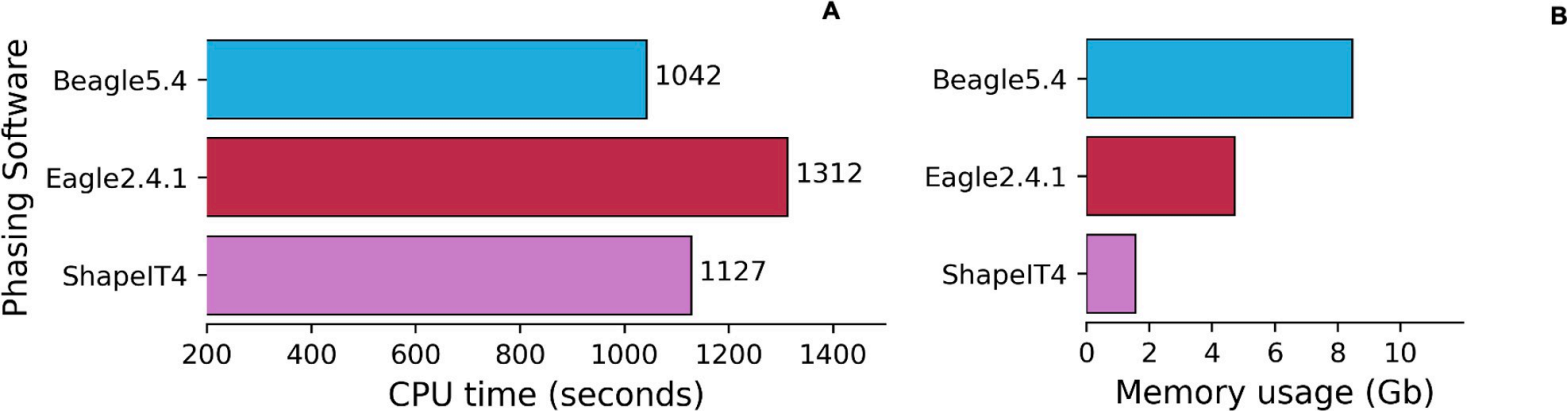
CPU run time and memory usage for phasing softwares in EBB chip dataset. Average run time for phasing (6A). Average memory usage for phasing (6B) in chips data.

The EBB chip dataset required 4x more memory usage and 4.7x more CPU time compared to the other 3 datasets. The increased number of samples highlighted differences between the tools with respect to computational efficiency in phasing. With smaller datasets, where the number of individuals was low, Eagle2.4.1 was the slowest phasing tool Fig 6A, but as the size of the dataset increased (2280 instead of 190), Shapeit4 required increasingly greater runtime for phasing, exceeding the run time of Beagle5.4 (Fig 7A).

**Fig 8:**
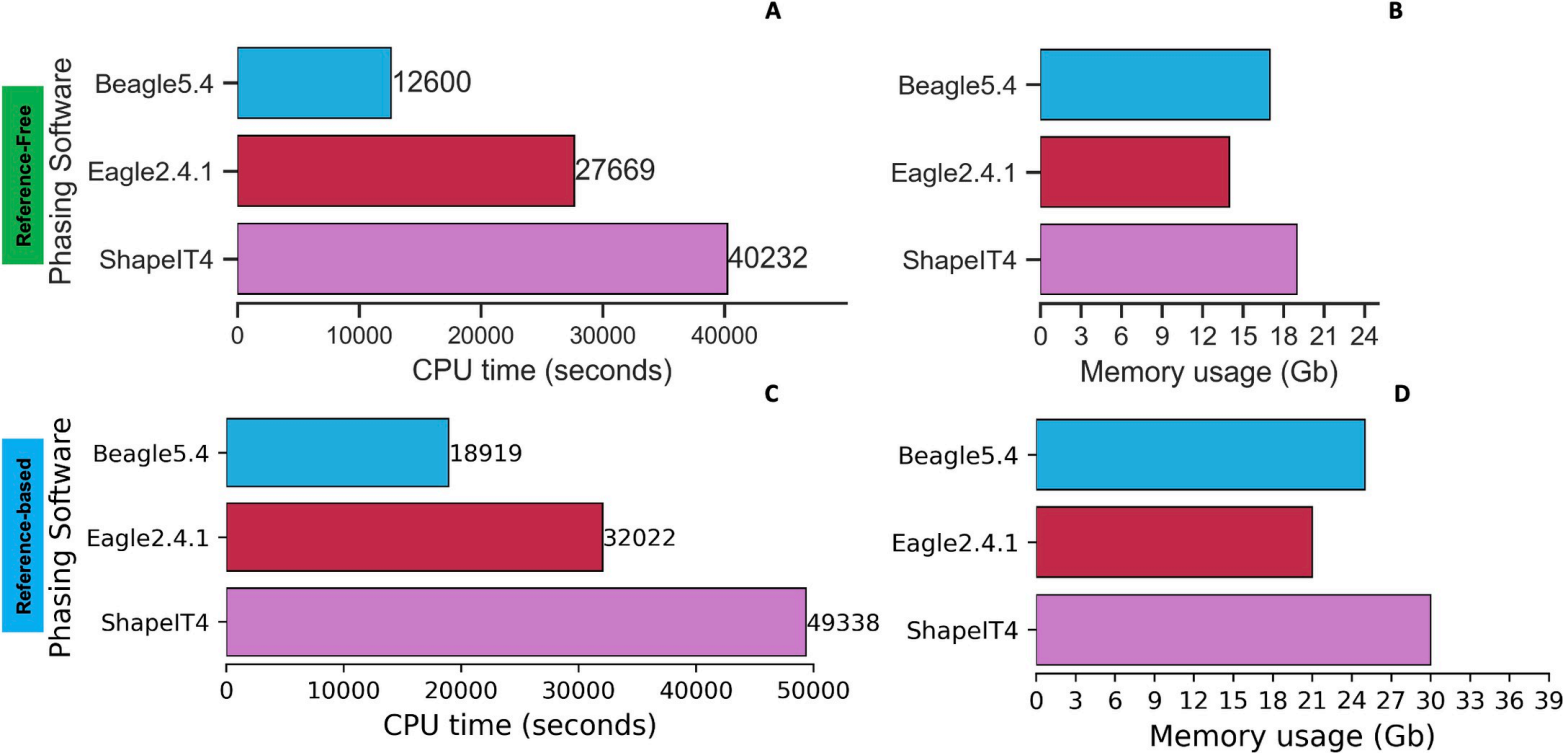
CPU run time and memory usage of phasing software in EBB WGS dataset. ShapeIT CPU time and memory usage are higher with a bigger input data of variants and individuals. (8A-8B) highlights a reference-free approach while (8C-8D) a reference-based approach.

We were also interested to see how these phasing softwares dealt with a bigger number of variants; thus, we used the WGS EBB dataset to include an additional whole genome sequencing phasing test to simulate a real-life scenario. We phased the entire WGS EBB dataset with 2280 individuals and ~1 million variants for chr20. The WGS Estonian Biobank dataset resulted in 3x more memory usage and 23x more CPU time compared to the EBB chip dataset (lower number of variants 13,990, same number of individuals 2280). We applied a reference-free and a reference-based approach using the entire 1000GP-30x data. With more variants and individuals, ShapeIT had higher CPU time (40232sec ~ 11.2h) (Fig 8A) and memory usage (19Gb) (Fig 8B) compared to a smaller dataset using a reference-free approach. Fig 8C and 8D show a reference-based approach.

### Imputation

#### Accuracy and Minor Allele Frequency (MAF) and reference panel

For the EBB chip data we stratified variants based on MAF and assessed imputation accuracy for common, infrequent, and rare variants to obtain a more nuanced understanding of how well each imputation tool performs (Table 3).

**Table 3:** MAF-stratified comparison of imputation software for EBB data.

MAF	Imputation Software	Sensitivity %	FPR %	#Variants
**MAF <5%**	**Beagle5.4**	99.538	1.252	72,493
	**Impute5**	99.546	1.272	72,493
	**Minimac4**	99.496	1.201	72,493
**MAF >5%**	**Beagle5.4**	97.951	3.282	429,884
	**Impute5**	97.911	3.303	429,884
	**Minimac4**	97.841	3.408	429,884

MAF<5% indicates all the variants that are below or equal to 5% in minor allele frequencies and MAF>5% indicates all the variants above 5% in minor allele frequencies. A comparison of the sensitivity and false positive rate (FPR) of the imputation results, for each phasing-imputation combination, stratified in two MAF categories.

**Fig 9:**
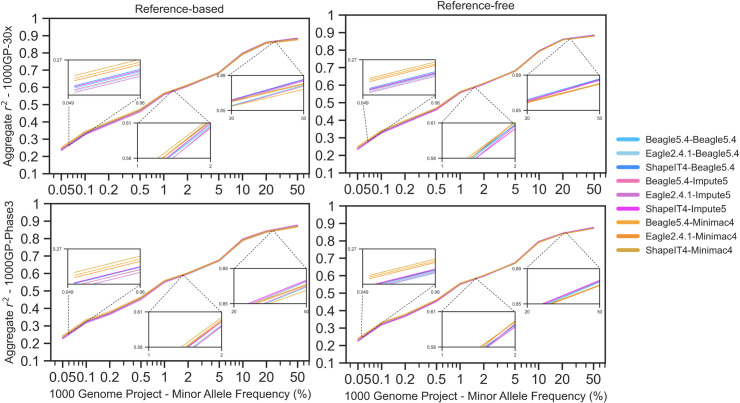
Imputation performance for chromosome 20 using EBB data with 2280 individuals with 2 reference panels and 2 phasing approaches. Blue colors indicate Beagle5.4, violets indicate Impute5 and oranges indicate Minimac4.

Based on the accuracy metric, the False Positive Rate (FPR), and the sensitivity, Beagle5.4 outperformed other imputation tools when MAF was greater than 5%, with Impute5 a close second. However, for uncommon variants (MAF≤5%), Minimac4 was the better imputation tool, with the lower FPR. Similar results were obtained using R^2^ as the metric (Fig 9). When it comes to reference panels, only with shared variants in common, 1000GP-30x has conducted to slightly higher results in accuracy, compared to 1000GP-Phase3. The slope of the curve was always higher for 1000GP-30x (Fig 9). The best phasing and imputation tool combination was ShapeIT4-Minimac4 using EBB chip with reference-based phasing and 1000GP-30x reference panel, resulting in an average imputation R^2^ of 0.536. Slightly worse results were obtained for 1000GP-Phase3 with an average imputation R^2^ of 0.527 for the same combination tested (S1 Table).

When using Affymetrix, Omni and customized chips the best combination overall was ShapeIT4-Beagle5.4 imputed from the Omni chip dataset (S4 Fig), with a reference-based phasing approach, and using the 1000GP-Phase3 reference panel, resulting in an average imputation R^2^ of 0.839 (S1 Table). The usage of 1000GP-Phase3 brings better results in terms of R^2^ imputation accuracy compared to the results gained with the 1000GP-30x reference panel in the same chip data when we discarded rare variants singletons and doubletons. On the other hand, for the 1000GP-30x reference panel, the best phasing and imputation tool combination was ShapeIT4-Impute5 using an Omni chip with reference-based phasing, resulting in an average imputation R^2^ of 0.728 (S1 Table).

**Fig 10:**
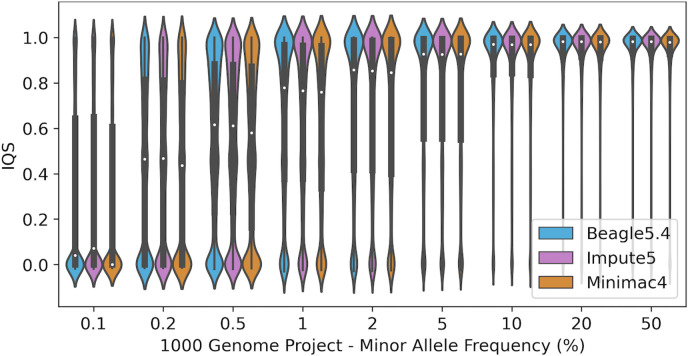
Evaluation of rare variants imputation. Violin plot of IQS against minor allele frequency (MAF) in the EBB dataset.

A good alternative metric to R^2^ is IQS. Fig 10 depicts an increase in IQS with increasing MAF. Impute5 produced better results at lower MAF than either Beagle5.4 or Minimac4, while Beagle5.4 imputed better above 5% allele frequency. Ultra-rare variants were imputed badly with all available software. A similar trend was also observed in Affymetrix, Omni and customized chip data (S5 Fig).

**Fig 11:**
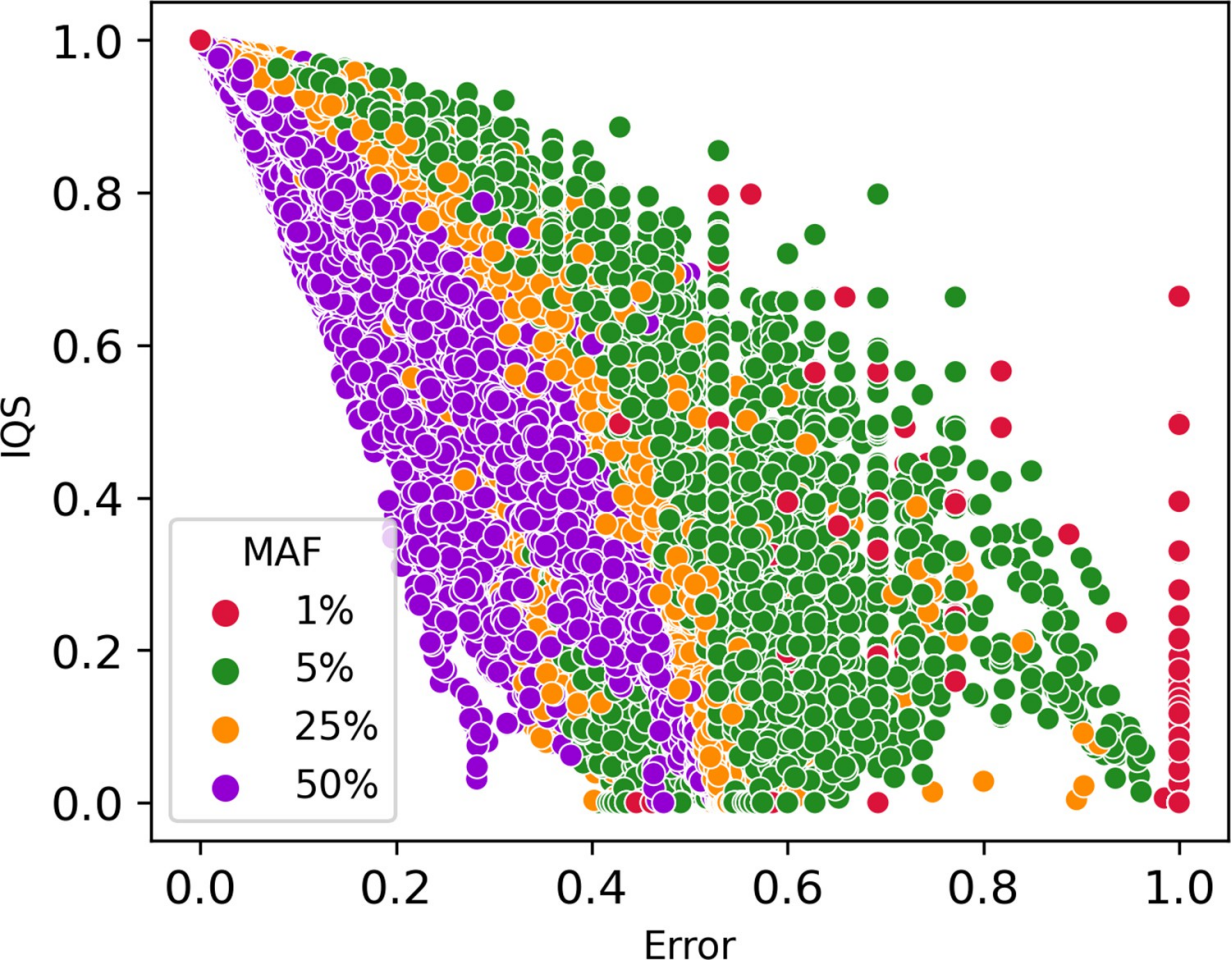
Minor allele frequency (MAF) stratification of imputed variants. Dots are clustered following minor allele frequency stratification. The dots clustered in the right-down corner of the figure have low IQS and high error rate, while dots in the left-high corner have high IQS and low error rate. Each dot represents the average IQS and error rate for a specific marker imputed with one phasing tool-imputation tool combination.

To get a better overall representation of how MAF affects imputation accuracy and error rates, we plotted IQS against the Error rate (Fig 11), where each dot represents an imputed variant. The markers clustered according to their MAF and followed a waterfall trend. The results of this analysis are shown in Fig 11, which illustrates that IQS is generally higher and error rates overall lower for more common variants. Rare variants, with MAF<1%, tend to have lower IQS and higher error rates.

#### The effect of phasing software choice on imputation accuracy

In EBB chip data choosing ShapeIT4 as the phasing tool for reference-based phasing, followed by any choice of imputation tool, resulted in the highest R^2^ for either imputation reference panel (S1 Table). For the Affymetrix and customized chips, ShapeIT4 remained the best choice of phasing tool for reference-free phasing, with respect to R^2^; for Omni, Beagle5.4 was the superior phasing tool. However, when we instead considered IQS as the metric of choice, both Beagle5.4 and ShapeIT4 performed equally well for reference-based phasing for higher density input chip datasets, but ShapeIT4 outperformed Beagle5.4 for the customized chip dataset, which had low chip density. For reference-free phasing, with respect to IQS, there was no clear winner between ShapeIT4 and Beagle5.4 (S1 Table). If we consider Concordance as a metric of choice, the Reference panel 1000GP-30x is the best choice to get higher imputation accuracy in every combination.

#### Population, sex, chip density, and phasing approach

**Fig 12:**
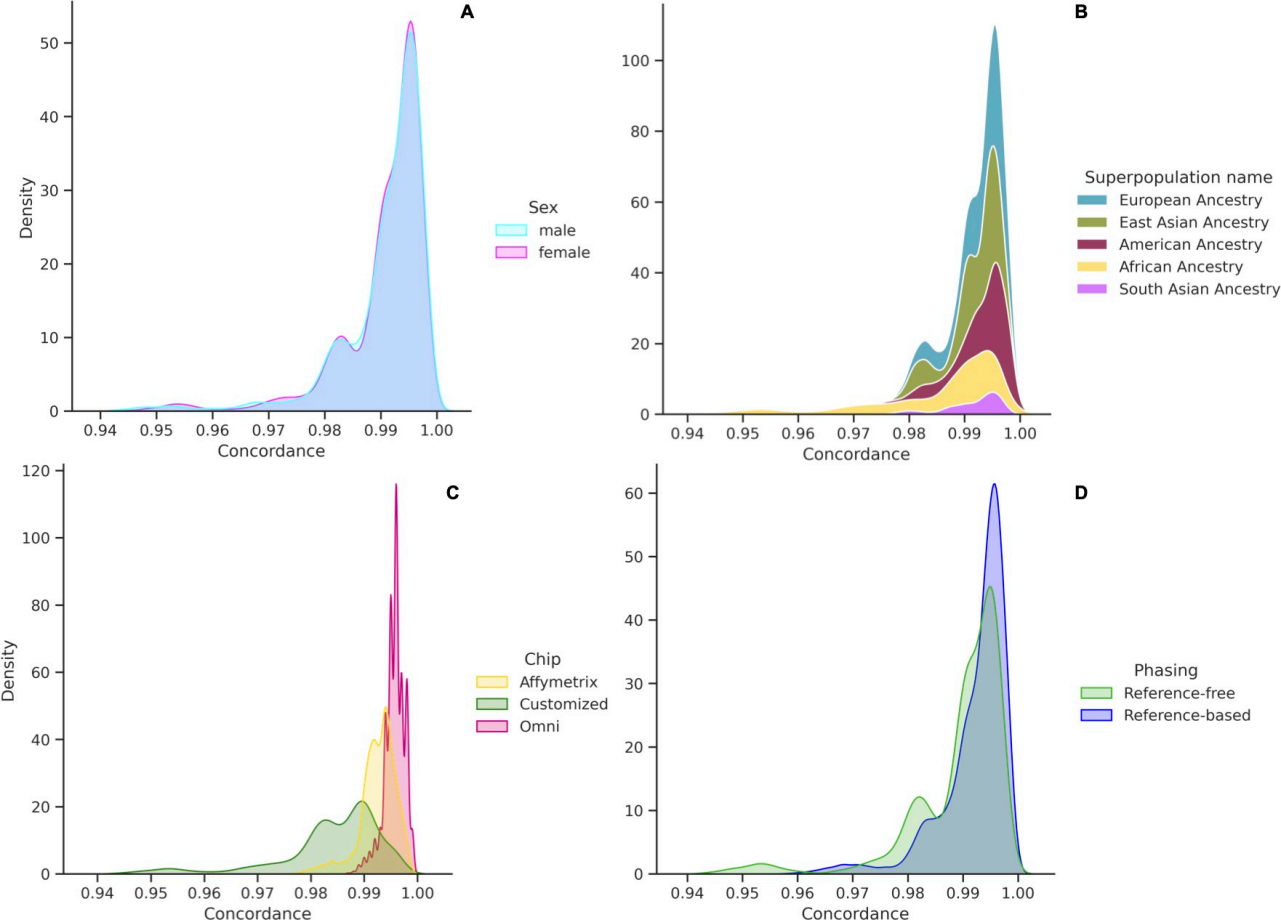
Imputation concordance rate over four different features. Stacked density plot of accuracy stratified by (A) sex; (B) superpopulation; (C) chip data; (D) phasing type (reference-free and reference-based).

Accuracy as measured by concordance (Po) was fairly close across superpopulations, with small differences between frameworks (Table 4, Fig 12). However, the mean imputation accuracy was lowest in individuals of African ancestry, and highest in individuals of European and American populations—groups which both have significant recent European ancestry (Table 4). Furthermore, despite reaching similar average imputation accuracy, a greater proportion of EUR individuals had very high imputation accuracy compared with a progressively smaller proportion of target individuals with higher concordance for East Asian, American, African and South Asian ancestry, respectively (Fig 12B). Thus, although we were able to reach similar mean imputation concordance for each of the different populations, imputation tools performed the best when applied to EUR populations and the worst for AFR and South Asian populations.

**Table 4:** Accuracy for different superpopulations in chips Affymetrix, Omni, Customized. Accuracy as measured by concordance (Po) of the imputation results for each of the five main super populations.

Superpopulation name	Mean	Std	#Individuals
**African**	0.984396	0.012613	40
**American**	0.993112	0.005104	40
**East Asian**	0.991575	0.004868	50
**European**	0.99274	0.004655	50
**South Asian**	0.991464	0.004989	10

**Fig 13:**
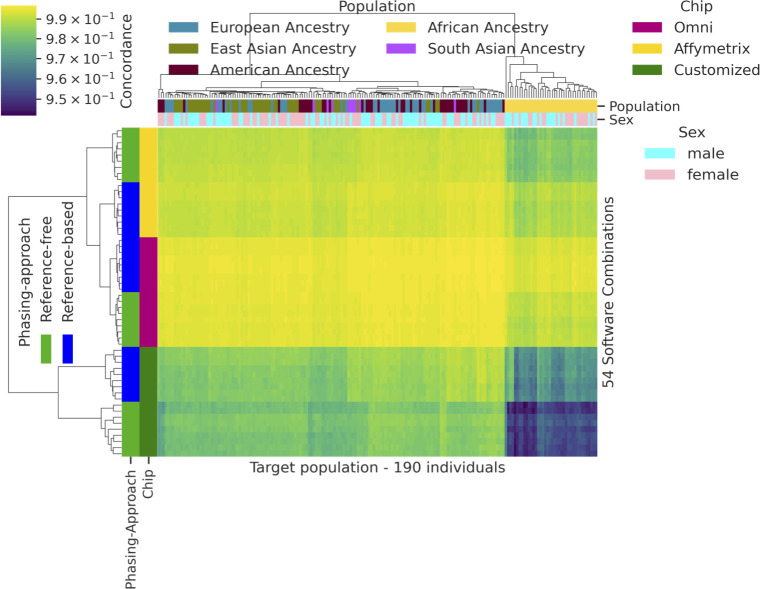
Cluster map of target population against 54 software-reference panel-dataset combinations. This figure depicts the concordance results for the reference-free and reference-based phasing approaches for each of these combinations. Higher density chips with a reference-based phasing approach and with populations without African ancestry obtained better results in terms of imputation accuracy measured by Concordance.

Differences in imputation accuracy by population and phasing approach are shown in Fig 12. The reference-based approach produced better results than the reference-free approach, for most combinations of imputation and phasing algorithms, based on a comparison of IQS across all combinations (Fig 12D). There was also a clear relationship between chip density and imputation accuracy, as measured by concordance; as chip density increased, imputation accuracy improved. The Omni chip had the greatest chip density and accuracy and the customized chip the lowest (Figs 12C and 13). From the shape of the chip distributions, we see that the vast majority of the Omni dataset was imputed with very high concordance, whereas less of the Affymetrix input dataset and much less of the Customized chip dataset was imputed with similar accuracy. We also compared imputation accuracy by sex as a check to ensure our QC process does not introduce any artificial differences. Sex had no effect on imputation accuracy for autosomal chromosome 20 (Fig 12A). Accuracy for females was on average 0.9907 ± 0.0078 while for males it was 0.9906 ± 0.0080.

#### Speed and memory usage in imputation

**Fig 14:**
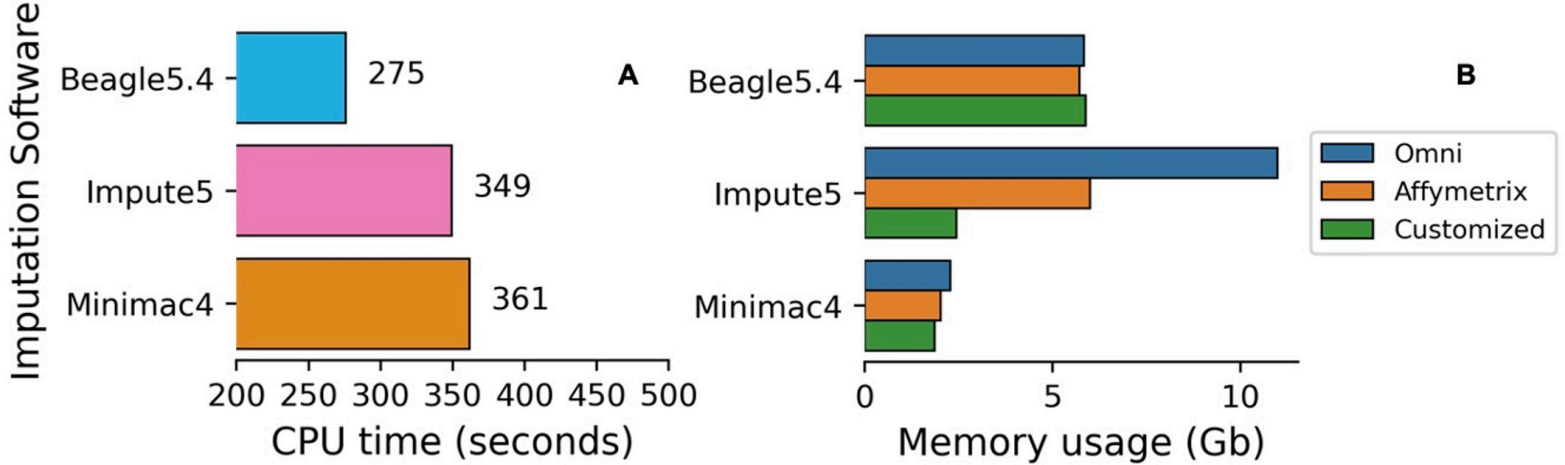
CPU run time and memory usage of imputation software for Affymetrix, Omni, Customized datasets. Average run time for imputation (A) tools. Average memory usage for imputation (B) tools in chips dataset.

**Fig 15:**
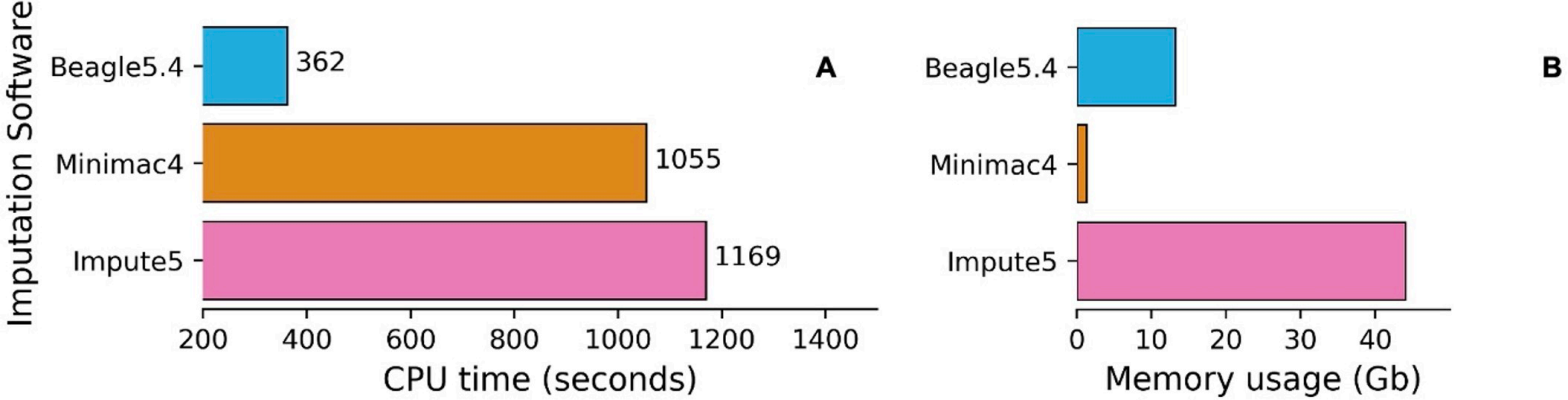
CPU run time and memory usage of imputation software for EBB chip data. Average run time for imputation (A) tools. Average memory usage for imputation (B) tools in EBB chip data.

Of the imputation software’s, Minimac4 appeared to be the most computationally efficient in terms of memory but had the slowest run time, followed by Beagle5.4 and Impute5 using chip data Affymetrix, Omni, Customized (Fig 14B). Memory usage for Impute5 increased drastically with the size of the input dataset used (EBB chip data with 2280 individuals), while Beagle5.4 and Minimac4 were not significantly affected (Fig 15). Beagle5.4 had the shortest run time, followed by Impute5 and Minimac4 (Fig 14A). Fig 15 shows the average computational run time for each combination. Phasing with ShapeIT4 and imputing with Beagle5.4 was the fastest combination, while phasing with Eagle2.4.1 and imputing with Minimac4 was the slowest.

**Fig 16:**
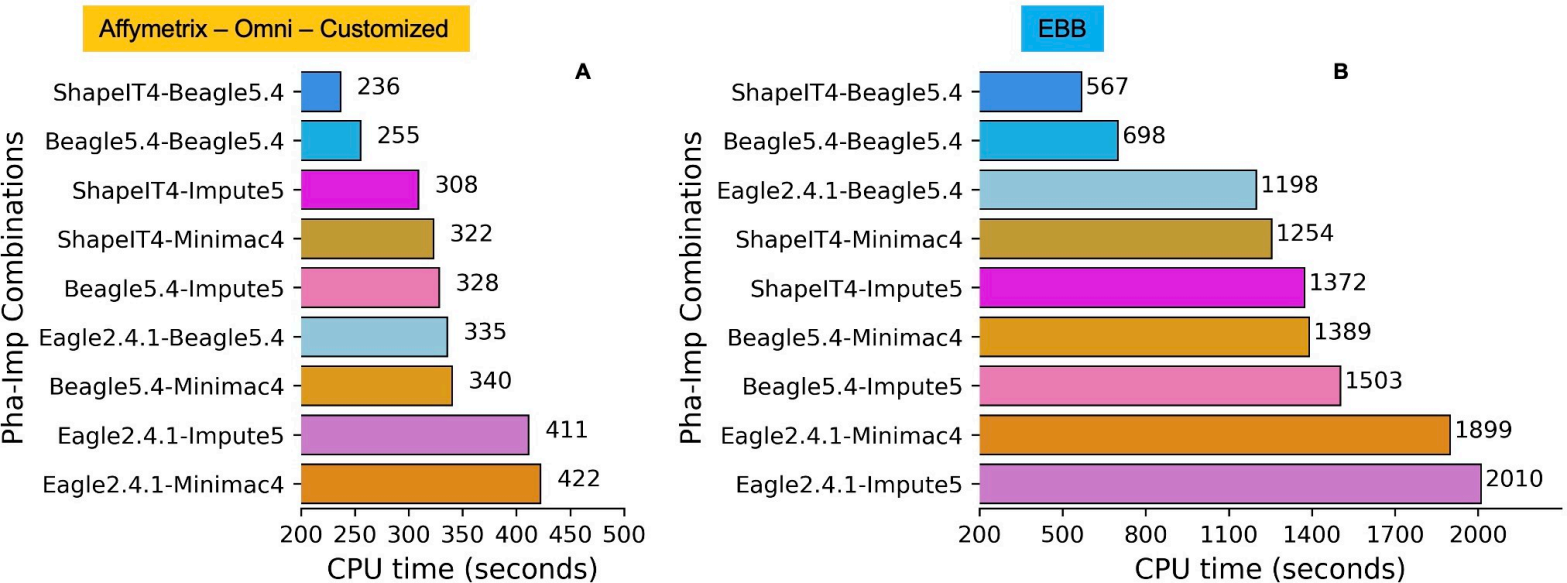
CPU run time of imputation and phasing combinations tested. Average run time for each of the 9 phasing and imputation software combinations. (A) Run time comparison of each combination in Affymetrix, Omni, Customized datasets. (B) Run time comparison of each combination in the EBB dataset.

Minimac4’s remained the most computationally efficient with regard to memory usage for both large and small sample sizes and Beagle5.4 continued to be fastest, but Impute5’s run time and memory usage increased exponentially with increased sample size, in absence of chunking (Fig 15). Fig 16A shows the average computational run time for each combination, while Fig 16B shows the differences in computation highlighted by the increase in sample size. Phasing with ShapeIT4 and imputing with Beagle5.4 remained the fastest combination, while phasing with Eagle2.4.1 and imputing with Impute5 dropped below the Eagle-Minimac combination to become the slowest with 3x more CPU time.

## Discussion

We performed a rigorous comparison of the most popular phasing and imputation tools currently used by genomics research groups to examine how the process of genotype imputation is affected by different factors, including the choice of reference panel, population, chip density, and allele frequency. We also compared the computational load of different datasets, tools and software combinations.

### Factors affecting imputation accuracy

Imputation accuracy decreased with chip density; the Affymetrix chip resulted in lower accuracy than the Omni chip and the customized chip had the lowest imputation accuracy. While this was expected, it also shows how our processing and comparison pipeline may help researchers design better chips by choosing the number and distribution of SNPs for each specific population and assessing the impact of density and SNP choice on phasing and imputation accuracy; it can also be used to determine whether different sets of chips are likely to perform better with certain combinations of phasing and imputation tools.

Next, we assessed both reference-free and reference-based phasing. Although reference-free phasing was less accurate than reference-based phasing with a reference panel containing admixed populations, increasing chip density reduced the degree of difference in phasing accuracy caused by the lack of reference. The difference between reference-free and reference-based phasing was small, suggesting that reference-free phasing may be acceptable in the absence of a representative reference panel. Further, the reference-free approach was more accurate when the reference panel populations did not match the target sample populations well. Similarly, previous studies comparing phasing accuracy with and without the use of a reference panel have shown that reference-free phasing, such as with Eagle2.4.1, can even lead to higher accuracy in cases where the reference panel ancestry and populations do not well match the sample individuals [14].

Furthermore, the choice of the reference panel may affect imputation accuracy, across all imputation metrics utilized. Interestingly, during the imputation of chips Affymetrix, Omni, and Customized, we got slightly better results in terms of R^2^ imputation accuracy, using 1000GP-Phase3, compared to the results gained with the 1000GP-30x reference panel in the same chip data when we discarded rare variants singletons and doubletons. This was due to the panel 1000GP-30x had more rare SNPs and the fact that R^2^ and IQS are heavily affected by the degree of uncertainty due to the rare SNPs. Indeed, if we look at the concordance rate, we will notice that concordance is higher (S1 Table) compared to the 1000GP-Phase3 reference panel suggesting that R^2^ and IQS are affected by rare SNPs (present only in the 1000GP-30x for Chip data Affymetrix, Omni, Customized), but this doesn’t happen to the overall accuracy that is higher in reference panel 1000GP-30x with the same combinations of tools and phasing approach (S1 Table). To check if the reference panel 1000GP-Phase3 was better for imputation, all the analyses with the EBB data, instead, have been conducted using both 1000GP-30x and 1000GP-Phase3 reference panels only with shared variants in common. We found higher values of R^2^ and IQS imputation accuracy for 1000GP-30x compared to the 1000GP-Phase3 reference panel, in all combinations tested. This suggests that the removal of variants singletons and doubletons increase the values of R^2^ and IQS but does not increase the imputation accuracy itself; this is a practice that should be avoided by scientists to prevent inflating the imputation accuracy results assessed with R^2^.

However, the use of concordance can also be confounding, as shown in Table 3. Sensitivity and False Positive Rate (FPR) are based on concordance rate; they are heavily affected by the number of variants that we are looking at, while R^2^ and IQS are less sensitive to these changes and in this case will better highlight the overall accuracy. MAF<5% appears to be higher in concordance compared to the MAF>5%, because the vast majority of the variants will be imputed correctly as homozygous reference and only few samples will have heterozygous or homozygous variants imputed wrongly.

Accuracy was further affected by population but not by sex using autosomal chromosomes. Different populations are characterized by differences in LD as a result of differences in genealogical history, and thus have different characteristic LD blocks and LD block sizes, which affect imputation accuracy [41]. We presume that lower imputation accuracy seen in individuals of AFR ancestry is attributable to the smaller LD blocks characteristic of AFR ancestry, which make it more difficult to correctly impute genotypes.

In agreement with previous research [42], we found that variants with low allele frequency are generally imputed poorly. In general, imputation works poorly for variants with low MAF as a function of both bias in the reference panels and bias in the software [42]. We can address reference-associated bias by significantly increasing the size of the chosen reference panel and including sufficient population-specific samples in the reference. However, addressing software bias would require developing improved imputation algorithms.

Finally, the choice of statistics is important when examining the imputation accuracy of rare and low frequency variants. We found that IQS and R^2^ produced similar means and standard deviations, though this does not necessarily represent similarity of values for particular SNPs. For rare and low frequency variants, concordance rates produce inflated assessments of accuracy [43] but reflect the real overall evaluation of an imputation software. The higher concordance rate values could mislead a researcher into assuming that these variants were imputed well. However, accuracy for less common variants is best measured using IQS and R^2^ [32].

### Choice of phasing and imputation tools

There was a discrepancy in accuracy based on different metrics. Highest average concordance rate was achieved by Beagle5.4 at 0.986, followed by Impute5 and Minimac4, using a reference-based approach during phasing, with the highest density chip dataset as input. In general, choosing Beagle5.4 for imputation and ShapeIT4 for phasing tended to get highly accurate results and was computationally faster even in larger datasets. When looking to improve the imputation of rare variants, however, researchers may want to use a mix of Beagle5.4, Impute5 and Minimac4 by applying Beagle5.4 to common variants and Minimac4, Impute5 to rare ones. Minimac4 and Impute5 tended to perform better on rare variants, because unlike Beagle5.4, which computes clusters of haplotypes and does its calculations based on those, Impute5 and Minimac4 search the whole space of haplotypes. This is more effective when imputing uncommon variants, but there is a tradeoff of increased computational load.

On the other hand, we see imputation accuracy for Beagle5.4 was better than Impute5 for the filtered phase3 reference panel; this was expected since the phase 3 panel has fewer rare alleles. Beagle5.4 was also the most stable tool to use across different input sizes. Minimac4 required the least amount of memory but took more time, which can be a good tradeoff depending on the purpose of the imputation. If the memory usage is limited, and the loss of accuracy is acceptable, then Minimac4 may be the optimal choice of imputation software. It is also important to note that the default parameters have been used for all software. For example, we could reduce the computational load of Impute5 by using parallel processing, but this could negatively affect the accuracy results; this negative impact was sufficient to reduce Impute5’s accuracy to below that of Beagle5.4 (data not shown). In conclusion, Beagle5.4 might have the best tradeoff between imputation quality and computational efficiency.

In closing, knowing the differences in imputation and phasing performance may prove useful in choosing imputation and phasing tools, depending on the intended downstream usage of the imputed results. However, this study also highlights that current tools are not accurate enough to impute rare and ultra-rare variants, showing that, when corrected for chance concordance and MAF bias, they result only in acceptable imputation accuracy and that there is significant scope for improvement.

## Supporting information

S1 Table Comparison of all combinations of phasing and imputation tool, reference panel, phasing approach, and chip datasets used in this study. All 144 combinations of phasing software, reference-based/reference-free phasing, imputation software, imputation reference panel, and input dataset, compared across the three accuracy metrics, concordance, R^2^, and IQS. The ranking/ordering is by R^2^ as it attempts to correct for MAF-bias and is a commonly used metric for imputation accuracy.
(DOCX)

S1 Fig Chip data used to assess imputation and phasing accuracy and origin of the customized chip. Affymetrix, Omni and Customized chips. SNP numbers for chromosome 20 are shown. Customized chip data was obtained from the intersection of the first two chips with the GSA chip.
(TIF)

S2 Fig Shared individuals between HD genotype chips and reference panels. Individuals in common between the WGS Reference panels, Omni and Affymetrix chips.
(TIF)

S3 Fig Origin of the target samples in chip data Affymetrix and Omni. Sample of 190 individuals belonging to 19 populations from 5 super populations selected for this study.
(TIF)

S4 Fig Imputation performance for chromosome 20 using 190 mixed population individuals with 2 reference panels and 2 phasing approaches. (TIF)

S5 Fig Evaluation of rare variants imputation. Violin plot. IQS is plotted against Minor allele frequency (MAF) for dataset Omni, Affymetrix and Customized.
(TIF)
