# Supplementary material for: A comparative analysis of current phasing and imputation software
Source: PLoS One. 2022 Oct 19;17(10):e0260177. doi: 10.1371/journal.pone.0260177 (PMC9581364; doi:10.1371/journal.pone.0260177)
Supplement: S1 Table — All 144 combinations of phasing software, reference-based/reference-free phasing, imputation software, imputation reference panel, and input dataset, compared across the three accuracy metrics, concordance, R2, and IQS. The ranking/ordering is by R2 as it attempts to correct for MAF-bias and is a commonly used metric for imputation accuracy. (DOCX) [file pone.0260177.s001.docx]

# **Supporting Information**

**S1 Table.** Comparison of all combinations of phasing and imputation tool, reference panel, phasing approach, and chip datasets used in this study. All 144 combinations of phasing software, reference-based/reference-free phasing, imputation software, imputation reference panel, and input dataset, compared across the three accuracy metrics, concordance, R^2^, and IQS. The ranking/ordering is by R^2^ as it attempts to correct for MAF-bias and is a commonly used metric for imputation accuracy.

| **Reference** | **Chip** | **Phasing** | **Combination** | **Concordance** | **r2** | **IQS** |
| --- | --- | --- | --- | --- | --- | --- |
| **1000GP-Phase3** | **Omni** | **Reference-based** | **ShapeIT4-Beagle5.4** | **0.993** | **0.839** | **0.818** |
| **1000GP-Phase3** | **Omni** | **Reference-based** | **Beagle5.4-Beagle5.4** | **0.993** | **0.838** | **0.816** |
| **1000GP-Phase3** | **Omni** | **Reference-based** | **Eagle2.4.1-Beagle5.4** | **0.993** | **0.836** | **0.813** |
| **1000GP-Phase3** | **Omni** | **Reference-based** | **Beagle5.4-Impute5** | **0.992** | **0.834** | **0.824** |
| **1000GP-Phase3** | **Omni** | **Reference-based** | **ShapeIT4-Impute5** | **0.992** | **0.832** | **0.824** |
| **1000GP-Phase3** | **Omni** | **Reference-based** | **Eagle2.4.1-Impute5** | **0.992** | **0.832** | **0.822** |
| **1000GP-Phase3** | **Omni** | **Reference-based** | **ShapeIT4-Minimac4** | **0.992** | **0.832** | **0.804** |
| **1000GP-Phase3** | **Omni** | **Reference-based** | **Beagle5.4-Minimac4** | **0.992** | **0.83** | **0.804** |
| **1000GP-Phase3** | **Omni** | **Reference-based** | **Eagle2.4.1-Minimac4** | **0.992** | **0.829** | **0.803** |
| **1000GP-Phase3** | **Omni** | **Reference-free** | **Beagle5.4-Impute5** | **0.99** | **0.787** | **0.742** |
| **1000GP-Phase3** | **Omni** | **Reference-free** | **Beagle5.4-Minimac4** | **0.99** | **0.785** | **0.725** |
| **1000GP-Phase3** | **Omni** | **Reference-free** | **ShapeIT4-Impute5** | **0.99** | **0.781** | **0.743** |
| **1000GP-Phase3** | **Omni** | **Reference-free** | **Beagle5.4-Beagle5.4** | **0.991** | **0.781** | **0.716** |
| **1000GP-Phase3** | **Affymetrix** | **Reference-based** | **ShapeIT4-Beagle5.4** | **0.988** | **0.78** | **0.741** |
| **1000GP-Phase3** | **Omni** | **Reference-free** | **ShapeIT4-Minimac4** | **0.99** | **0.78** | **0.726** |
| **1000GP-Phase3** | **Affymetrix** | **Reference-based** | **Beagle5.4-Beagle5.4** | **0.988** | **0.779** | **0.743** |
| **1000GP-Phase3** | **Omni** | **Reference-free** | **ShapeIT4-Beagle5.4** | **0.991** | **0.778** | **0.726** |
| **1000GP-Phase3** | **Affymetrix** | **Reference-based** | **Eagle2.4.1-Beagle5.4** | **0.988** | **0.776** | **0.739** |
| **1000GP-Phase3** | **Omni** | **Reference-free** | **Eagle2.4.1-Impute5** | **0.99** | **0.773** | **0.723** |
| **1000GP-Phase3** | **Omni** | **Reference-free** | **Eagle2.4.1-Beagle5.4** | **0.991** | **0.77** | **0.703** |
| **1000GP-Phase3** | **Affymetrix** | **Reference-based** | **Beagle5.4-Impute5** | **0.987** | **0.769** | **0.758** |
| **1000GP-Phase3** | **Affymetrix** | **Reference-based** | **ShapeIT4-Impute5** | **0.987** | **0.768** | **0.755** |
| **1000GP-Phase3** | **Omni** | **Reference-free** | **Eagle2.4.1-Minimac4** | **0.99** | **0.768** | **0.707** |
| **1000GP-Phase3** | **Affymetrix** | **Reference-based** | **ShapeIT4-Minimac4** | **0.987** | **0.768** | **0.732** |
| **1000GP-Phase3** | **Affymetrix** | **Reference-based** | **Beagle5.4-Minimac4** | **0.987** | **0.768** | **0.735** |
| **1000GP-Phase3** | **Affymetrix** | **Reference-based** | **Eagle2.4.1-Impute5** | **0.987** | **0.765** | **0.754** |
| **1000GP-Phase3** | **Affymetrix** | **Reference-based** | **Eagle2.4.1-Minimac4** | **0.987** | **0.761** | **0.73** |
| **1000GP-30x** | **Omni** | **Reference-based** | **ShapeIT4-Impute5** | **0.994** | **0.728** | **0.746** |
| **1000GP-30x** | **Omni** | **Reference-based** | **Beagle5.4-Impute5** | **0.994** | **0.727** | **0.745** |
| **1000GP-30x** | **Omni** | **Reference-based** | **ShapeIT4-Beagle5.4** | **0.994** | **0.724** | **0.742** |
| **1000GP-30x** | **Omni** | **Reference-based** | **Beagle5.4-Beagle5.4** | **0.994** | **0.723** | **0.74** |
| **1000GP-30x** | **Omni** | **Reference-based** | **Eagle2.4.1-Impute5** | **0.994** | **0.723** | **0.742** |
| **1000GP-30x** | **Omni** | **Reference-based** | **Eagle2.4.1-Beagle5.4** | **0.994** | **0.718** | **0.736** |
| **1000GP-Phase3** | **Affymetrix** | **Reference-free** | **ShapeIT4-Beagle5.4** | **0.986** | **0.704** | **0.631** |
| **1000GP-Phase3** | **Affymetrix** | **Reference-free** | **Beagle5.4-Impute5** | **0.984** | **0.699** | **0.658** |
| **1000GP-30x** | **Omni** | **Reference-based** | **ShapeIT4-Minimac4** | **0.993** | **0.698** | **0.714** |
| **1000GP-Phase3** | **Affymetrix** | **Reference-free** | **ShapeIT4-Minimac4** | **0.984** | **0.697** | **0.633** |
| **1000GP-Phase3** | **Affymetrix** | **Reference-free** | **ShapeIT4-Impute5** | **0.984** | **0.697** | **0.659** |
| **1000GP-Phase3** | **Affymetrix** | **Reference-free** | **Beagle5.4-Minimac4** | **0.984** | **0.697** | **0.633** |
| **1000GP-Phase3** | **Affymetrix** | **Reference-free** | **Beagle5.4-Beagle5.4** | **0.985** | **0.696** | **0.631** |
| **1000GP-30x** | **Omni** | **Reference-based** | **Beagle5.4-Minimac4** | **0.993** | **0.696** | **0.712** |
| **1000GP-30x** | **Omni** | **Reference-based** | **Eagle2.4.1-Minimac4** | **0.992** | **0.692** | **0.708** |
| **1000GP-30x** | **Affymetrix** | **Reference-based** | **ShapeIT4-Impute5** | **0.991** | **0.687** | **0.713** |
| **1000GP-30x** | **Affymetrix** | **Reference-based** | **Beagle5.4-Impute5** | **0.991** | **0.685** | **0.711** |
| **1000GP-30x** | **Affymetrix** | **Reference-based** | **ShapeIT4-Beagle5.4** | **0.992** | **0.682** | **0.706** |
| **1000GP-Phase3** | **Affymetrix** | **Reference-free** | **Eagle2.4.1-Minimac4** | **0.983** | **0.681** | **0.617** |
| **1000GP-Phase3** | **Affymetrix** | **Reference-free** | **Eagle2.4.1-Beagle5.4** | **0.985** | **0.68** | **0.606** |
| **1000GP-30x** | **Affymetrix** | **Reference-based** | **Beagle5.4-Beagle5.4** | **0.992** | **0.68** | **0.703** |
| **1000GP-30x** | **Affymetrix** | **Reference-based** | **Eagle2.4.1-Impute5** | **0.991** | **0.679** | **0.706** |
| **1000GP-Phase3** | **Affymetrix** | **Reference-free** | **Eagle2.4.1-Impute5** | **0.983** | **0.679** | **0.641** |
| **1000GP-30x** | **Affymetrix** | **Reference-based** | **Eagle2.4.1-Beagle5.4** | **0.991** | **0.673** | **0.698** |
| **1000GP-30x** | **Omni** | **Reference-free** | **ShapeIT4-Impute5** | **0.993** | **0.656** | **0.678** |
| **1000GP-30x** | **Affymetrix** | **Reference-based** | **ShapeIT4-Minimac4** | **0.99** | **0.654** | **0.677** |
| **1000GP-Phase3** | **Customized** | **Reference-based** | **ShapeIT4-Beagle5.4** | **0.978** | **0.652** | **0.608** |
| **1000GP-30x** | **Affymetrix** | **Reference-based** | **Beagle5.4-Minimac4** | **0.99** | **0.652** | **0.675** |
| **1000GP-30x** | **Omni** | **Reference-free** | **Beagle5.4-Impute5** | **0.992** | **0.652** | **0.675** |
| **1000GP-30x** | **Affymetrix** | **Reference-based** | **Eagle2.4.1-Minimac4** | **0.99** | **0.646** | **0.669** |
| **1000GP-30x** | **Omni** | **Reference-free** | **Eagle2.4.1-Impute5** | **0.992** | **0.64** | **0.664** |
| **1000GP-Phase3** | **Customized** | **Reference-based** | **Eagle2.4.1-Beagle5.4** | **0.977** | **0.637** | **0.591** |
| **1000GP-30x** | **Omni** | **Reference-free** | **ShapeIT4-Beagle5.4** | **0.993** | **0.636** | **0.657** |
| **1000GP-Phase3** | **Customized** | **Reference-based** | **Beagle5.4-Beagle5.4** | **0.977** | **0.636** | **0.593** |
| **1000GP-30x** | **Omni** | **Reference-free** | **Beagle5.4-Beagle5.4** | **0.993** | **0.634** | **0.656** |
| **1000GP-Phase3** | **Customized** | **Reference-based** | **ShapeIT4-Minimac4** | **0.975** | **0.634** | **0.604** |
| **1000GP-Phase3** | **Customized** | **Reference-based** | **ShapeIT4-Impute5** | **0.975** | **0.628** | **0.641** |
| **1000GP-Phase3** | **Customized** | **Reference-based** | **Beagle5.4-Minimac4** | **0.975** | **0.624** | **0.586** |
| **1000GP-Phase3** | **Customized** | **Reference-based** | **Eagle2.4.1-Minimac4** | **0.974** | **0.62** | **0.588** |
| **1000GP-Phase3** | **Customized** | **Reference-based** | **Eagle2.4.1-Impute5** | **0.975** | **0.62** | **0.63** |
| **1000GP-30x** | **Omni** | **Reference-free** | **Eagle2.4.1-Beagle5.4** | **0.992** | **0.619** | **0.642** |
| **1000GP-30x** | **Omni** | **Reference-free** | **ShapeIT4-Minimac4** | **0.991** | **0.619** | **0.638** |
| **1000GP-Phase3** | **Customized** | **Reference-based** | **Beagle5.4-Impute5** | **0.975** | **0.619** | **0.625** |
| **1000GP-30x** | **Omni** | **Reference-free** | **Beagle5.4-Minimac4** | **0.991** | **0.617** | **0.637** |
| **1000GP-30x** | **Omni** | **Reference-free** | **Eagle2.4.1-Minimac4** | **0.991** | **0.604** | **0.625** |
| **1000GP-30x** | **Customized** | **Reference-based** | **ShapeIT4-Impute5** | **0.982** | **0.592** | **0.638** |
| **1000GP-30x** | **Customized** | **Reference-based** | **ShapeIT4-Beagle5.4** | **0.984** | **0.589** | **0.629** |
| **1000GP-30x** | **Customized** | **Reference-based** | **Beagle5.4-Impute5** | **0.982** | **0.586** | **0.632** |
| **1000GP-30x** | **Customized** | **Reference-based** | **Beagle5.4-Beagle5.4** | **0.984** | **0.581** | **0.621** |
| **1000GP-30x** | **Customized** | **Reference-based** | **Eagle2.4.1-Impute5** | **0.982** | **0.581** | **0.628** |
| **1000GP-30x** | **Affymetrix** | **Reference-free** | **ShapeIT4-Impute5** | **0.988** | **0.58** | **0.613** |
| **1000GP-30x** | **Customized** | **Reference-based** | **Eagle2.4.1-Beagle5.4** | **0.983** | **0.576** | **0.617** |
| **1000GP-30x** | **Affymetrix** | **Reference-free** | **Beagle5.4-Impute5** | **0.987** | **0.575** | **0.608** |
| **1000GP-30x** | **Affymetrix** | **Reference-free** | **Eagle2.4.1-Impute5** | **0.987** | **0.561** | **0.596** |
| **1000GP-30x** | **Affymetrix** | **Reference-free** | **ShapeIT4-Beagle5.4** | **0.988** | **0.557** | **0.586** |
| **1000GP-30x** | **Affymetrix** | **Reference-free** | **Beagle5.4-Beagle5.4** | **0.988** | **0.553** | **0.583** |
| **1000GP-30x** | **Affymetrix** | **Reference-free** | **ShapeIT4-Minimac4** | **0.986** | **0.54** | **0.568** |
| **1000GP-30x** | **Customized** | **Reference-based** | **ShapeIT4-Minimac4** | **0.98** | **0.539** | **0.578** |
| **1000GP-30x** | **EBB** | **Reference-based** | **ShapeIT4-Minimac4** | **0.968** | **0.538** | **0.586** |
| **1000GP-30x** | **Affymetrix** | **Reference-free** | **Eagle2.4.1-Beagle5.4** | **0.987** | **0.536** | **0.568** |
| **1000GP-30x** | **Affymetrix** | **Reference-free** | **Beagle5.4-Minimac4** | **0.986** | **0.535** | **0.564** |
| **1000GP-30x** | **EBB** | **Reference-free** | **ShapeIT4-Minimac4** | **0.968** | **0.535** | **0.581** |
| **1000GP-30x** | **EBB** | **Reference-based** | **Eagle2.4.1-Minimac4** | **0.968** | **0.534** | **0.584** |
| **1000GP-30x** | **EBB** | **Reference-based** | **ShapeIT4-Beagle5.4** | **0.968** | **0.534** | **0.596** |
| **1000GP-30x** | **Customized** | **Reference-based** | **Beagle5.4-Minimac4** | **0.98** | **0.533** | **0.572** |
| **1000GP-30x** | **EBB** | **Reference-based** | **Beagle5.4-Minimac4** | **0.967** | **0.533** | **0.582** |
| **1000GP-30x** | **EBB** | **Reference-free** | **Beagle5.4-Minimac4** | **0.968** | **0.533** | **0.579** |
| **1000GP-30x** | **EBB** | **Reference-free** | **Eagle2.4.1-Minimac4** | **0.968** | **0.533** | **0.578** |
| **1000GP-30x** | **EBB** | **Reference-based** | **ShapeIT4-Impute5** | **0.968** | **0.532** | **0.597** |
| **1000GP-30x** | **EBB** | **Reference-free** | **ShapeIT4-Beagle5.4** | **0.968** | **0.532** | **0.592** |
| **1000GP-30x** | **EBB** | **Reference-based** | **Eagle2.4.1-Beagle5.4** | **0.968** | **0.53** | **0.593** |
| **1000GP-30x** | **EBB** | **Reference-free** | **Beagle5.4-Beagle5.4** | **0.968** | **0.53** | **0.59** |
| **1000GP-30x** | **EBB** | **Reference-free** | **ShapeIT4-Impute5** | **0.968** | **0.53** | **0.593** |
| **1000GP-30x** | **Customized** | **Reference-based** | **Eagle2.4.1-Minimac4** | **0.98** | **0.529** | **0.57** |
| **1000GP-30x** | **EBB** | **Reference-based** | **Beagle5.4-Beagle5.4** | **0.968** | **0.529** | **0.591** |
| **1000GP-30x** | **EBB** | **Reference-free** | **Eagle2.4.1-Beagle5.4** | **0.968** | **0.529** | **0.59** |
| **1000GP-30x** | **EBB** | **Reference-free** | **Beagle5.4-Impute5** | **0.968** | **0.529** | **0.591** |
| **1000GP-30x** | **EBB** | **Reference-free** | **Eagle2.4.1-Impute5** | **0.968** | **0.529** | **0.59** |
| **1000GP-30x** | **EBB** | **Reference-based** | **Eagle2.4.1-Impute5** | **0.968** | **0.528** | **0.593** |
| **1000GP-30x** | **EBB** | **Reference-based** | **Beagle5.4-Impute5** | **0.968** | **0.528** | **0.591** |
| **1000GP-Phase3** | **EBB** | **Reference-based** | **ShapeIT4-Minimac4** | **0.958** | **0.527** | **0.576** |
| **1000GP-Phase3** | **EBB** | **Reference-free** | **ShapeIT4-Minimac4** | **0.959** | **0.526** | **0.572** |
| **1000GP-Phase3** | **EBB** | **Reference-based** | **Eagle2.4.1-Minimac4** | **0.958** | **0.525** | **0.575** |
| **1000GP-Phase3** | **EBB** | **Reference-based** | **ShapeIT4-Impute5** | **0.959** | **0.524** | **0.586** |
| **1000GP-Phase3** | **EBB** | **Reference-based** | **Beagle5.4-Minimac4** | **0.958** | **0.524** | **0.572** |
| **1000GP-Phase3** | **EBB** | **Reference-based** | **ShapeIT4-Beagle5.4** | **0.959** | **0.524** | **0.588** |
| **1000GP-Phase3** | **EBB** | **Reference-free** | **Beagle5.4-Minimac4** | **0.958** | **0.524** | **0.569** |
| **1000GP-Phase3** | **EBB** | **Reference-free** | **Eagle2.4.1-Minimac4** | **0.959** | **0.524** | **0.569** |
| **1000GP-Phase3** | **EBB** | **Reference-free** | **ShapeIT4-Impute5** | **0.959** | **0.522** | **0.582** |
| **1000GP-Phase3** | **EBB** | **Reference-free** | **ShapeIT4-Beagle5.4** | **0.96** | **0.522** | **0.582** |
| **1000GP-Phase3** | **EBB** | **Reference-free** | **Beagle5.4-Impute5** | **0.959** | **0.521** | **0.583** |
| **1000GP-Phase3** | **EBB** | **Reference-free** | **Eagle2.4.1-Impute5** | **0.959** | **0.521** | **0.583** |
| **1000GP-Phase3** | **EBB** | **Reference-based** | **Eagle2.4.1-Impute5** | **0.958** | **0.52** | **0.581** |
| **1000GP-Phase3** | **EBB** | **Reference-based** | **Eagle2.4.1-Beagle5.4** | **0.959** | **0.52** | **0.581** |
| **1000GP-Phase3** | **EBB** | **Reference-based** | **Beagle5.4-Impute5** | **0.958** | **0.52** | **0.581** |
| **1000GP-Phase3** | **EBB** | **Reference-free** | **Beagle5.4-Beagle5.4** | **0.959** | **0.52** | **0.581** |
| **1000GP-Phase3** | **EBB** | **Reference-based** | **Beagle5.4-Beagle5.4** | **0.959** | **0.519** | **0.583** |
| **1000GP-Phase3** | **EBB** | **Reference-free** | **Eagle2.4.1-Beagle5.4** | **0.959** | **0.519** | **0.584** |
| **1000GP-30x** | **Affymetrix** | **Reference-free** | **Eagle2.4.1-Minimac4** | **0.986** | **0.518** | **0.548** |
| **1000GP-Phase3** | **Customized** | **Reference-free** | **ShapeIT4-Beagle5.4** | **0.97** | **0.509** | **0.43** |
| **1000GP-Phase3** | **Customized** | **Reference-free** | **ShapeIT4-Minimac4** | **0.967** | **0.503** | **0.432** |
| **1000GP-Phase3** | **Customized** | **Reference-free** | **ShapeIT4-Impute5** | **0.967** | **0.494** | **0.484** |
| **1000GP-Phase3** | **Customized** | **Reference-free** | **Beagle5.4-Minimac4** | **0.967** | **0.494** | **0.429** |
| **1000GP-Phase3** | **Customized** | **Reference-free** | **Beagle5.4-Beagle5.4** | **0.97** | **0.492** | **0.427** |
| **1000GP-Phase3** | **Customized** | **Reference-free** | **Beagle5.4-Impute5** | **0.967** | **0.486** | **0.48** |
| **1000GP-Phase3** | **Customized** | **Reference-free** | **Eagle2.4.1-Beagle5.4** | **0.968** | **0.479** | **0.417** |
| **1000GP-Phase3** | **Customized** | **Reference-free** | **Eagle2.4.1-Minimac4** | **0.965** | **0.471** | **0.401** |
| **1000GP-Phase3** | **Customized** | **Reference-free** | **Eagle2.4.1-Impute5** | **0.965** | **0.469** | **0.461** |
| **1000GP-30x** | **Customized** | **Reference-free** | **ShapeIT4-Impute5** | **0.972** | **0.404** | **0.46** |
| **1000GP-30x** | **Customized** | **Reference-free** | **Beagle5.4-Impute5** | **0.972** | **0.399** | **0.456** |
| **1000GP-30x** | **Customized** | **Reference-free** | **Eagle2.4.1-Impute5** | **0.97** | **0.374** | **0.431** |
| **1000GP-30x** | **Customized** | **Reference-free** | **ShapeIT4-Beagle5.4** | **0.974** | **0.367** | **0.414** |
| **1000GP-30x** | **Customized** | **Reference-free** | **Beagle5.4-Beagle5.4** | **0.973** | **0.362** | **0.409** |
| **1000GP-30x** | **Customized** | **Reference-free** | **Eagle2.4.1-Beagle5.4** | **0.972** | **0.335** | **0.383** |
| **1000GP-30x** | **Customized** | **Reference-free** | **ShapeIT4-Minimac4** | **0.97** | **0.334** | **0.377** |
| **1000GP-30x** | **Customized** | **Reference-free** | **Beagle5.4-Minimac4** | **0.97** | **0.328** | **0.372** |
| **1000GP-30x** | **Customized** | **Reference-free** | **Eagle2.4.1-Minimac4** | **0.968** | **0.303** | **0.347** |
