# Supplementary figures and images for: A comparative analysis of current phasing and imputation software
Source: PLoS One. 2022 Oct 19;17(10):e0260177. doi: 10.1371/journal.pone.0260177 (PMC9581364; doi:10.1371/journal.pone.0260177)

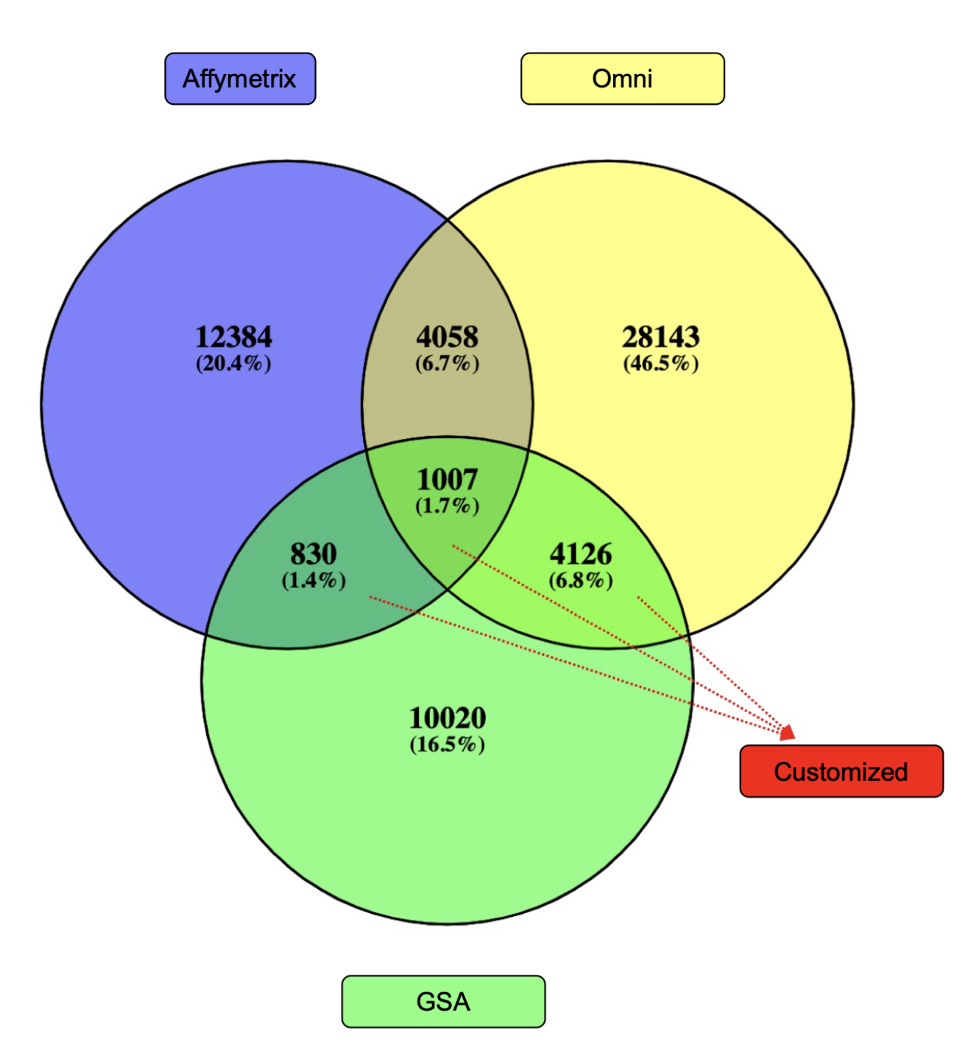

Supplement: S1 Fig — Affymetrix, Omni and Customized chips. SNP numbers for chromosome 20 are shown. Customized chip data was obtained from the intersection of the first two chips with the GSA chip. (TIF) [file pone.0260177.s002.tif]

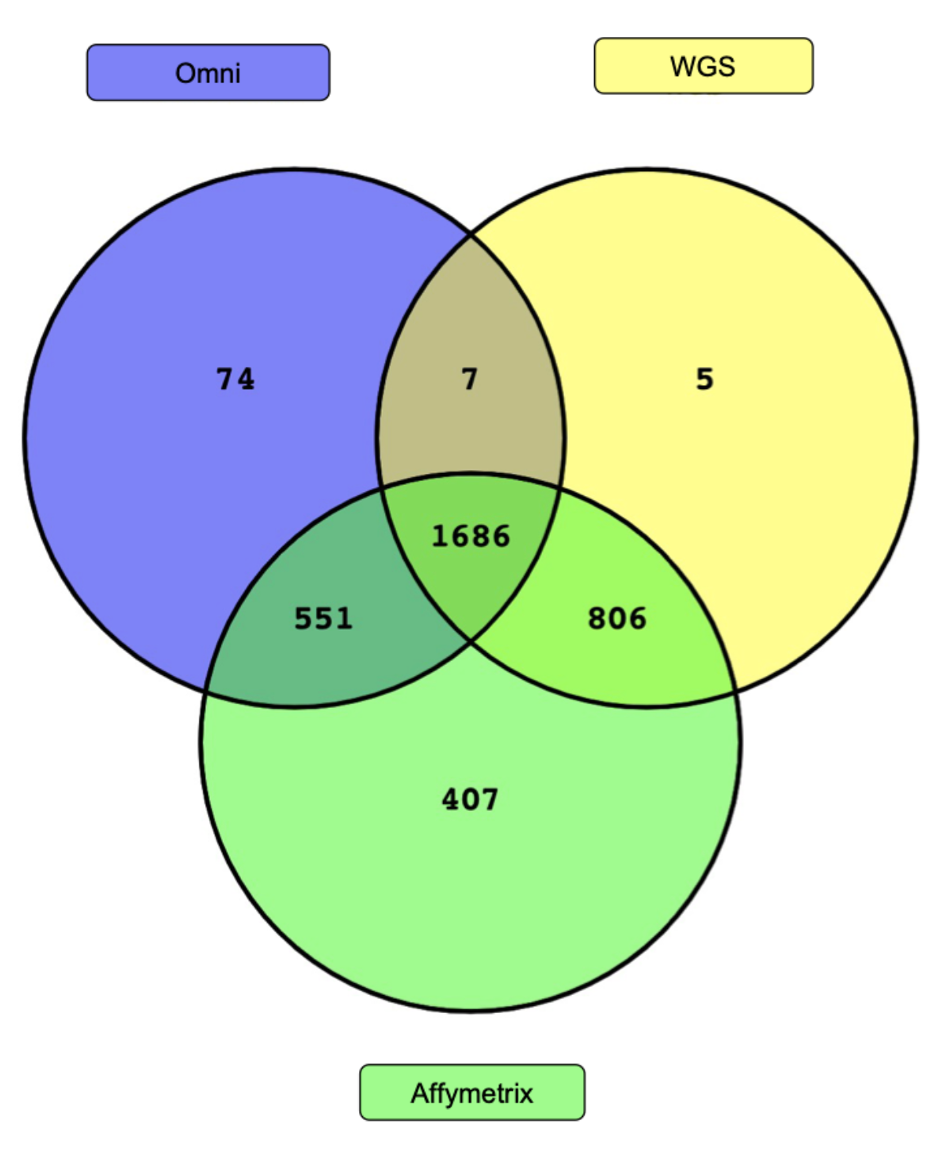

Supplement: S2 Fig — Individuals in common between the WGS Reference panels, Omni and Affymetrix chips. (TIF) [file pone.0260177.s003.tif]

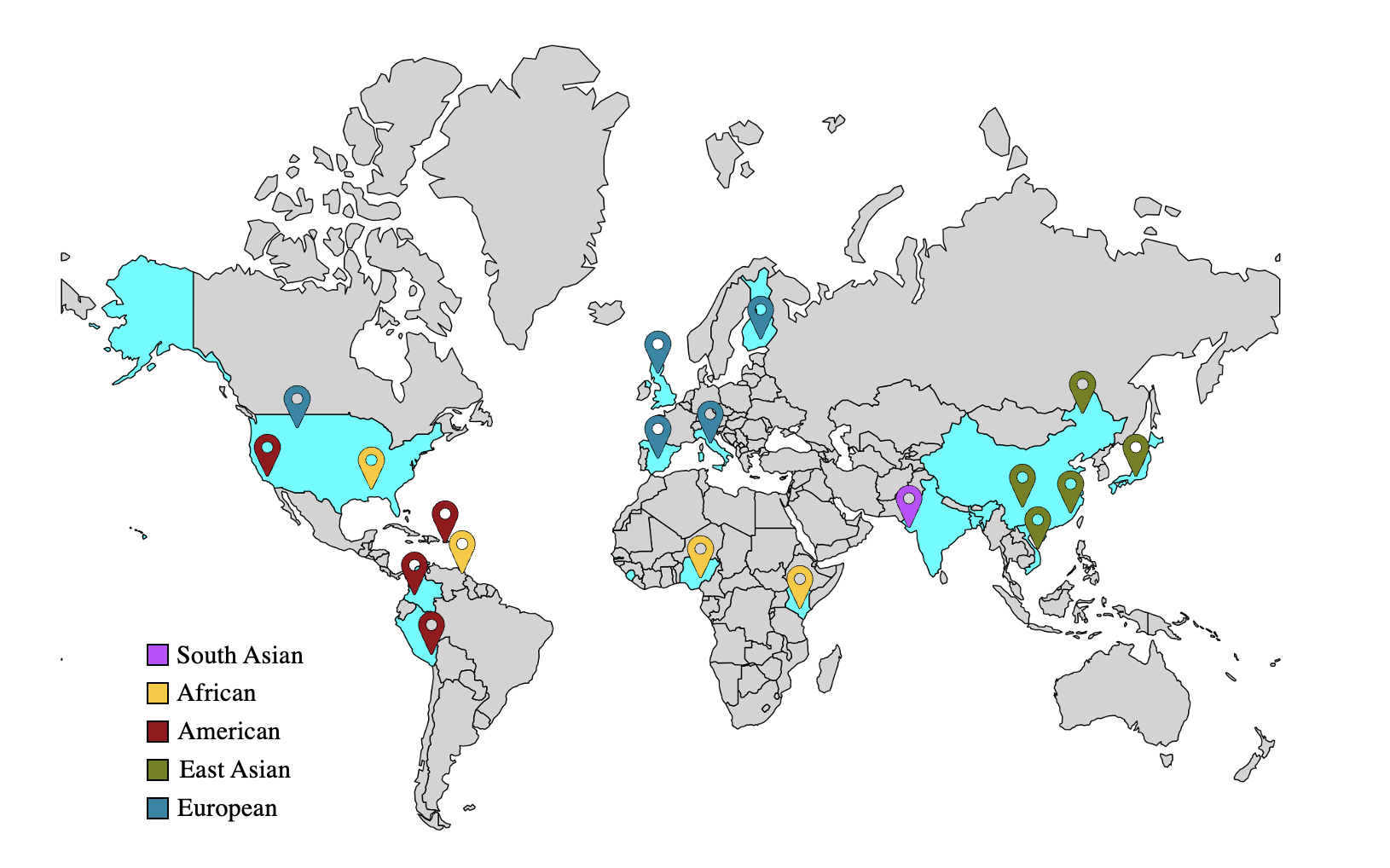

Supplement: S3 Fig — Sample of 190 individuals belonging to 19 populations from 5 super populations selected for this study. (TIF) [file pone.0260177.s004.tif]

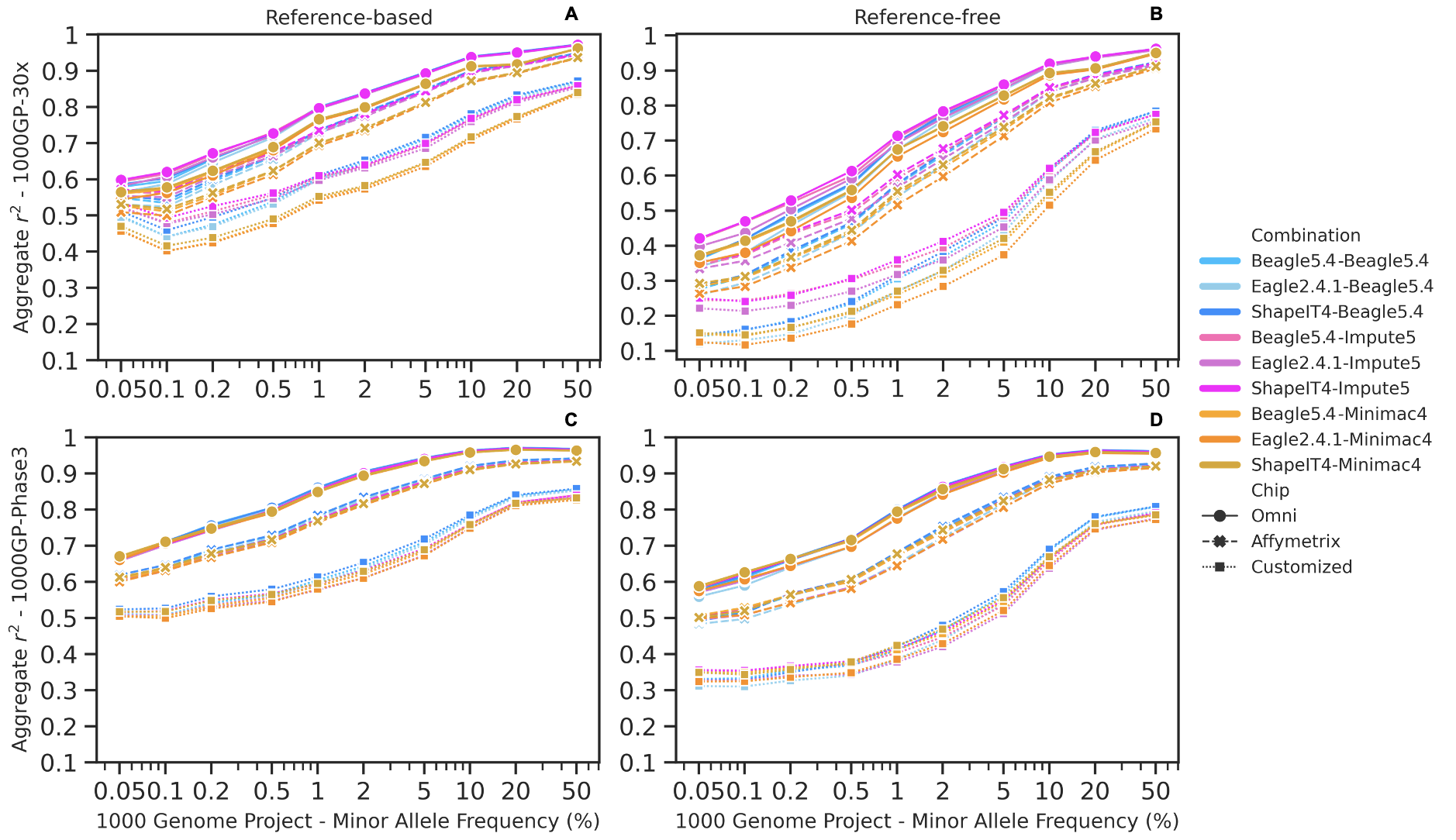

Supplement: S4 Fig — (TIF) [file pone.0260177.s005.tif]

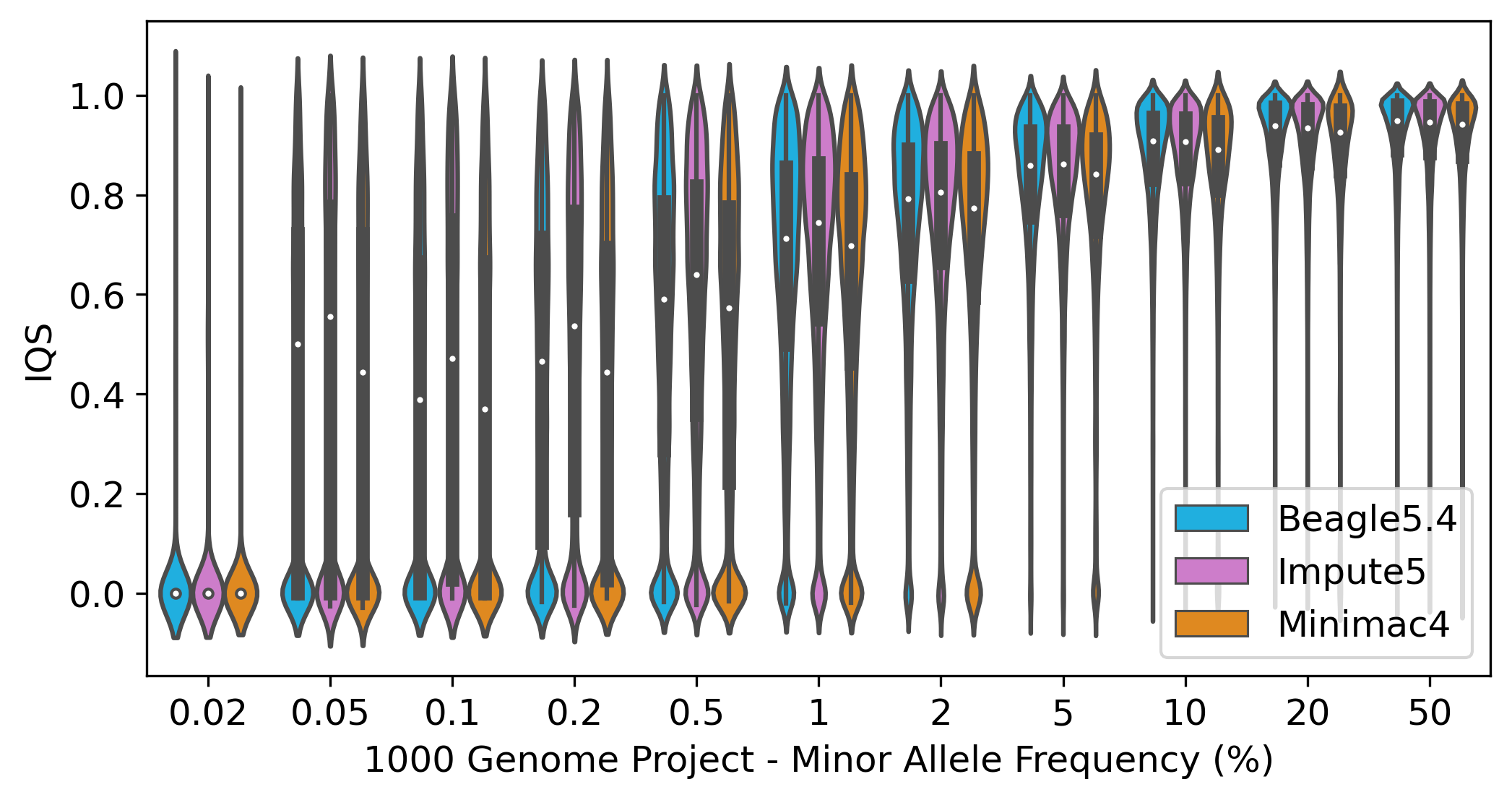

Supplement: S5 Fig — Violin plot. IQS is plotted against Minor allele frequency (MAF) for dataset Omni, Affymetrix and Customized. (TIF) [file pone.0260177.s006.tif]
